# Effectiveness of an expanded role for community health workers on malaria blood examination rates in malaria elimination settings in Myanmar: an open stepped-wedge, cluster-randomised controlled trial

**DOI:** 10.1016/j.lansea.2024.100499

**Published:** 2024-10-17

**Authors:** Win Han Oo, Win Htike, May Chan Oo, Pwint Phyu Phyu, Kyawt Mon Win, Nay Yi Yi Linn, Tun Min, Ei Phyu Htwe, Aung Khine Zaw, Kaung Myat Thu, Naw Hkawng Galau, Julia C. Cutts, Julie A. Simpson, Nick Scott, Katherine O’Flaherty, Paul A. Agius, Freya J.I. Fowkes

**Affiliations:** a Disease Elimination Program, Burnet Institute, 85 Commercial Road, Melbourne, VIC, Australia; b Centre for Epidemiology and Biostatistics, Melbourne School of Population and Global Health, the University of Melbourne, 207 Bouverie Street, Parkville, VIC 3053, Australia; c Health Security Program, Burnet Institute Myanmar, 226 U Wisara Road, Yangon, Myanmar; d Yangon Regional Public Health Department, Ministry of Health, 520 West Horse Race Course Road, Yangon, Myanmar; e National Malaria Control Programme, Department of Public Health, Ministry of Health, Zabu Kyetthayay Road, Nay Pyi Taw, Myanmar; f Faculty of Health, Deakin University, 221 Burwood Hwy, Melbourne, VIC, Australia; g Department of Epidemiology and Preventive Medicine, Monash University, 553 St Kilda Road, Melbourne, VIC 3004, Australia

**Keywords:** Community health worker, Malaria elimination, Primary health care, Myanmar

## Abstract

**Background:**

As Greater Mekong Subregion countries approach malaria elimination, the motivation and social role of community health workers (CHWs), and malaria blood examination rates, have declined in parallel with decreasing malaria burden. To address this issue, a health system model which expanded the role for CHWs was co-designed with communities and health stakeholders in the Mekong Subregion and field-tested in Myanmar.

**Methods:**

An open stepped-wedge cluster-randomised (at the village-level) controlled trial (ClinicalTrials.govNCT04695886) was conducted in 72 villages in Myanmar from Nov 1, 2021 to April 17, 2022 to evaluate the effectiveness and cost-effectiveness of the expanded CHW model. One-off and continuous implementation costs of the models were calculated.

**Findings:**

A total of 2886 malaria rapid diagnostic tests (RDTs) (control period: 1365; intervention period: 1521) were undertaken across 72 villages during the 24-week study period. Compared to the existing CHW model, the introduction of an expanded role for CHWs resulted in a 23% relative increase in village weekly malaria blood examination rates by RDT, the primary outcome, (adjusted incidence rate ratio (AIRR) = 1.23, 95% CI = 1.01, 1.50, p = 0.036), adjusting for time and season. A 3.3-fold relative increase in village weekly referral rate of dengue, tuberculosis, diarrhoea, or RDT-negative fever cases after the introduction of the expanded CHW model (AIRR = 3.17, 95% CI = 1.23, 8.18, p = 0.017), was also observed. The total cost per CHW per five-year period was US$14,794 for the expanded CHW model and $5816 for the existing CHW model.

**Interpretation:**

An expanded CHW model, co-designed with communities and health stakeholders, can increase malaria blood examination rates in malaria elimination settings and referral rates for other infectious diseases. Expanded CHW models will facilitate maintaining annual blood examination rates required for malaria elimination accreditation by the WHO.

**Funding:**

An International Multilateral Donor (QSE-M-UNOPS-BI-20864-007-40).

**Research in context**

**Evidence before this study**

We searched published articles in PubMed, Embase, Cochrane Central Register of Controlled Trials, LILACS, African Medicus Index and grey literature in Google Scholar and websites of national malaria programmes, UN agencies and international organisations using the keywords ((malaria OR plasmodium OR falciparum OR vivax) AND (health volunteer OR service provider OR staff OR community health worker OR basic health worker OR primary health care provider OR traditional birth attendan∗ OR auxiliary midwi∗ OR public health supervisor OR community intervention OR community model)) with no restrictions on language published up to Feb 5, 2019. We reviewed studies conducted in malaria-endemic areas that contain data describing the outcomes or processes involved in community-based delivery of any malaria or malaria plus other diseases interventions. We identified one systematic review which pooled results from 28 studies and quantified the positive impact of community-delivered models on intervention coverage and malariametric indices. Only three studies were performed in low transmission settings and no studies included in the review investigated the impact of community-delivered models on malaria blood examination rates. We also identified one study which demonstrated that the implementation of a community health worker model which delivered malaria and other services for common illnesses in Myanmar was associated with an increase in malaria blood examination rates. However, this study was an observational study undertaken in the malaria control not elimination phase and was not co-designed with end-users and stakeholders.

**Added value of this study**

This trial provides the highest quality of evidence to date that an expanded community health worker model, co-designed with communities and healthcare stakeholders, can increase the rates of malaria blood examination, as well as referral for other common infectious diseases, in a malaria elimination setting. Cost analyses quantified the additional costs of an expanded community health worker model.

**Implications of all the available evidence**

The implementation of an expanded role for community health workers in malaria elimination settings may maintain the annual blood examination rate required for malaria case detection and malaria elimination accreditation by the WHO. In addition, the provision of services by community health workers for common infectious diseases may facilitate the achievement of universal health coverage. As countries of the Greater Mekong Subregion, and others approaching malaria elimination, consider an expanded role of community health workers, this evidence-base provides impetus for the implementation of co-designed expanded community health worker models into country and regional malaria elimination, primary health care and universal health coverage policies.

## Introduction

The Greater Mekong Subregion (GMS), which includes Cambodia, China (Yunnan Province), Lao People’s Democratic Republic, Myanmar, Thailand and Vietnam, aims to eliminate malaria by 2030.^1^ Malaria elimination is a continuous process that needs context-specific intervention packages to meet national health systems. Importantly, in the malaria elimination phase, malaria surveillance should be transformed into a core intervention, and prevention, diagnosis and treatment interventions should be universally accessible.^2^ Malaria diagnosis using rapid diagnostic tests (RDTs) is crucial for surveillance and treatment of malaria in elimination and prevention of re-introduction phases. High annual blood examination rates for malaria are critical to detect and treat all malaria cases and are an essential requirement for WHO malaria elimination certification.^3^ Blood examination rates also need to be maintained at a high-rate post-elimination to detect and respond to imported cases so that elimination is maintained.

In the GMS, malaria volunteers, also known as malaria Community Health Workers (CHWs), played a critical role in reducing malaria cases and deaths and transitioning the region from control to elimination phases.^4^ Malaria CHWs provide essential malaria services, including malaria diagnosis using RDT in rural areas where the malaria burden is the highest and coverage of formal health services is limited. This network of malaria CHWs is expanding across the GMS because they can operate with minimal training and are effective and cost-effective in resource-limited countries.^4^, ^5^, ^6^, ^7^ However, as GMS countries approach malaria elimination, the motivation and social role of malaria CHWs,^8^, ^9^, ^10^ and malaria blood examination rates, have declined in parallel with the decreasing malaria burden.^11^ Consequently, GMS countries have been considering the expansion of malaria CHW roles (malaria plus other primary health care services)^12^^,^^13^ to maintain high malaria blood examination rates to detect and treat all malaria cases and ensure WHO malaria elimination certification.^3^

Observational studies integrating malaria services into expanded general health care during the malaria control phase, where the proportion of RDT testing positive for malaria is relatively high, have observed increased malaria blood examination rates compared to programs delivering services solely for malaria.^11^^,^^14^, ^15^, ^16^ However, the impact of expanded CHW services, typically providing malaria plus other primary health care services, in malaria elimination settings, where the annual parasite incidence of less than one may be different. Furthermore, these previous observational studies did not implement an expanded CHW model co-designed by end users, beneficiaries, and health stakeholders, including national programs, and therefore these models were not adopted as national policy. An expanded CHW model will have greatest impact if co-designed with the end users, beneficiaries, and stakeholders that is, CHWs, community members and malaria stakeholders. After extensive systematic reviews,^4^ and qualitative consultations (focus group discussions, semi-structured interviews, participatory workshops that applied participatory learning and action methods) engaging key stakeholders from the Ministry of Health, non-government implementing partners, current CHWs, and community leaders and members in Myanmar^8^^,^^9^ and Lao People’s Democratic Republic,^10^ findings were triangulated an expanded CHW model, the Community-delivered Integrated Malaria Elimination Model (the CIME model), was co-designed.^17^ The CIME model, which has yet to be field implemented, is specific for malaria elimination settings and offers services for malaria elimination, but also dengue, tuberculosis (TB), RDT-negative fever and childhood diarrhoea^17^ and addresses the specific needs of the end users and beneficiaries in the malaria elimination context where it will be implemented.

To determine the effectiveness of the co-designed CIME model compared to current CHW models being implemented in GMS elimination settings, we implemented the CIME model in an elimination setting in Myanmar, where the current malaria CHWs are known as Integrated Community Malaria Volunteers (ICMVs). Taking the number of weekly malaria blood examinations by RDTs undertaken by CHWs as the primary outcome, and referrals for dengue, TB, RDT-negative fever and childhood diarrhoea as secondary outcomes, the CIME model was evaluated in terms of its effectiveness, acceptability, fidelity, feasibility, and cost-effectiveness,^18^ compared to ICMV, to inform its use in primary health care in Myanmar and other malaria endemic, resource-limited countries approaching malaria elimination.^17^

## Methods

The study protocol (Supplementary Material S1) was published elsewhere^17^ and is summarised below.

### Study setting, design and participants

Since the 1980s, Myanmar has implemented a CHWs program for communicable disease control activities. With increased funding from international donors, the focus of CHWs shifted to malaria control and many CHWs became malaria CHWs in 2010–2011, providing services for malaria only.^19^^,^^20^ CHWs are volunteer providers and each CHWs works at their own pace, as per their specific personal circumstance and the level of demand from the village community they serve. CHWs typically use their house as the clinic. People present and receive services at the CHW residence passively. CHWs also perform active case detection of malaria in the community and provided mass education. In 2017–2018, as the malaria program was shifting from control to the elimination phase, malaria CHWs were trained to deliver control activities for an additional five diseases, namely dengue, HIV/AIDS, leprosy, lymphatic filariasis and TB^13^ and rebranded as ICMVs.

**Fig. 1:**
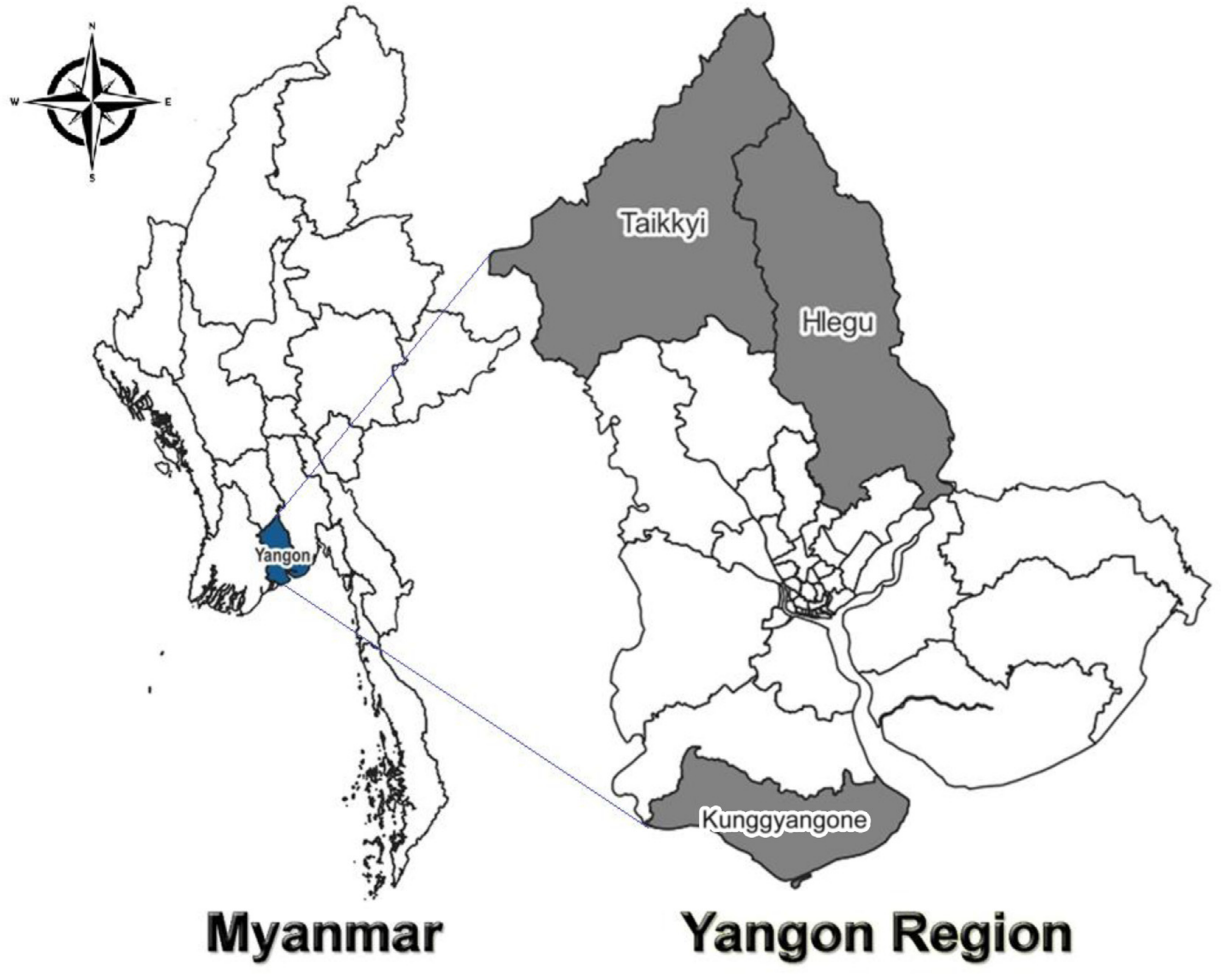
**Study townships of Hlegu, Kunggyangone and Taikkyi in Yangon Region, Myanmar**.

Between Nov 1, 2021 and April 17, 2022, a 24-week stepped-wedged cluster randomised trial (Supplementary Material S6—Fig. S1) was conducted in the three townships (31, 17 and 24 clusters (villages) in Hlegu, Kunggyangone and Taikkyi Townships, respectively) in Yangon, Myanmar (Fig. 1). The Yangon Region is a low malaria transmission area with annual parasite incidence of less than one over the past three consecutive years prior to the trial (2019–2021).^21^ As per the Myanmar National Malaria Elimination Plan, Yangon aims for subnational malaria elimination by 2025.^22^ The study townships were selected as per operational feasibility, security, and presence of malaria transmission and CHWs managed by Yangon Vector Borne Diseases Control Unit. All CHWs and their servicing villages in the selected three townships were eligible. The study was conducted over ‘cool’ (November–February) and ‘hot’ (March–April) seasonal periods.

All eligible participants were asked to read and sign the Participant Information and Consent Forms outlining the scope of the study, the risks and benefits of participating in the study procedures and their role in the study. Both paper-based and electronic data were maintained with utmost confidentiality. This study has been registered in ClinicalTrials.gov (Trial registration number–NCT04695886) and approved by the Institutional Review Board, Myanmar Department of Medical Research (Ethics/DMR/2020/111) and Alfred Hospital Ethics Review Committee, Australia (241/20).

### Randomisation and masking

**Fig. 2:**
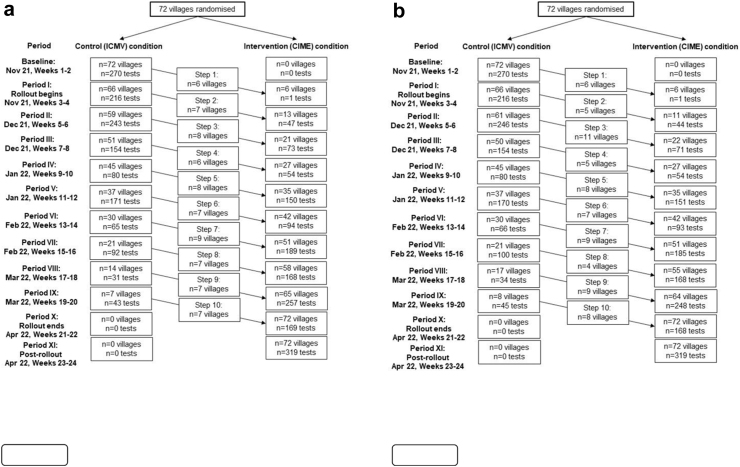
**The expanded Community Health Worker (CHW) models study flow-chart showing the number of villages and the number of malaria blood examinations by rapid diagnostic test (RDT) by study steps (2a: as per study protocol and 2b: as treated).** The Community-delivered Integrated Malaria Elimination (CIME) model intervention was implemented sequentially in 72 villages (clusters) serviced by 72 CIME volunteers. Note: a: The numbers of villages and tests observed at each ‘period’ of the study, outlined in this figure, are based on the a priori randomised sequences. Divergence in the number of villages transitioned into the intervention relative to the original unbalanced stepped-wedge study design was due to a second round of block randomisation of replacement villages (ICMVs) which was necessary to address initial village (ICMV) attrition or withdrawal, non-consent and contact failure. Tests undertaken during training periods are not included in analyses. b: The numbers of villages and tests observed at each ‘period’ of the study, outlined in this figure, are based on the observed as treated randomised sequences.

Using a computer-based block randomisation routine, the trial statistician randomised de-identified villages to respective ordered sequences to ensure the study week at which villages started receiving the intervention during the 24-week study period. The stepped-wedge study design was incomplete in type, such that villages were randomised into 10 ordered blocks/sequences in an unbalanced fashion (Supplementary Material S6—Fig. S1). While the study originally sought 140 villages for participation (detailed below), the final 72 included villages were randomised into the following sequences–seven villages in the first nine sequences and nine villages in a single sequence transitioned at the final step of the study. Blocks/sequences of villages were transitioned from control (CHW providing ICMV services) to intervention (CHWs providing CIME services) conditions at fortnightly intervals following a four-day training and transition period, beginning in November 2021. This followed an initial two-week baseline control period at the start of the study where all village clusters used the ICMV model exclusively (Fig. 2). Neither the participants, nor the implementors were blinded to the village sequence randomisation as it was not possible to blind CHWs from undergoing training prior to transitioning into the intervention condition.

### Procedures

In this study, the intervention is the co-designed CIME model for health service delivery. The CIME model involves CHWs delivering specific yet integrated services for malaria elimination, dengue, TB, RDT-negative fever and childhood diarrhoea; each CHW typically covered their residing village. Applying the Training Curriculum (Supplementary Material S2) which was designed to train the CHWs to provide CIME health services, CIME training was conducted at township health departments of the each of the three participating townships jointly by Burnet Institute Myanmar and Yangon Vector Borne Diseases Control unit staff, staged per the randomisation of the village sequences. Over the approximate four-days of training, medicines and commodities were distributed to CHWs to implement the CIME model. After training, CHWs commenced operating as CIME CHWs in their respective villages, and Yangon Vector Borne Diseases Control staff performed at least one supervision visit to each CHW and used the checklists to assess the CHW performance (Supplementary Material S3). During the three CIME data collection meetings held at the township health departments organised jointly by Burnet Institute Myanmar and Yangon Vector Borne Diseases Control Unit for the purpose of collecting implementation data on health services provision by CHWs, medicines and commodities were replenished for uninterrupted supply until the end of the trial period. During the study period, CHWs continued receiving the same amount in incentives (50,000 kyats [approximately 20 US$] per three months) after transitioning from applying ICMV to CIME health services.

In November 2021, baseline data such as geographic information, sociodemographic data and available malaria services in the participating villages were collected (Supplementary Material S4) from Yangon Vector Borne Diseases Control unit and CHWs. After the baseline period, implementation of the CIME model commenced and trial data were recorded using four different types of data collection formats (Supplementary Material S5), which were collected at data collection meetings held at township health departments. These data were then entered into Epidata 4.6 and subsequently imported into Stata version 17 along with baseline data.

### Outcomes

The primary outcome was the number of weekly blood examinations by CHWs undertaken for malaria using the Standard Diagnostic Bioline Malaria Ag *Plasmodium (P) falciparum/P*. *vivax* RDT. Secondary outcomes reported here include *Plasmodium* spp. infection detected by RDT, notified after diagnosis and treated as per the National Malaria Treatment Guidelines. Secondary outcomes also included weekly referral of suspected dengue, TB, diarrhoea or RDT-negative fever cases to health centres as well as cost-effectiveness of the CIME model.^17^

### Statistical analyses

Power estimation was based on the estimation of an intervention effect from a stepped-wedge cluster randomised design assuming analysis by Poisson mixed-effects modelling.^23^^,^^24^ Given the study design and assuming 140 CHW servicing villages from the original sampling frame, a baseline malaria RDT blood examination rate of approximately two tests per week per village, an estimate of between-village heterogeneity in testing (ICC (Intra Cluster Correlation)) of *ρ* = 0.30 (estimated from analyses of unpublished trial data),^25^ the study was estimated to be powered (90%) to detect a minimum 13% relative increase (rate ratio = 1.13) in testing attributable to the intervention (α = 5% (two-sided)). While the study originally sought 140 villages for participation, 72 villages were randomised in the final study.

To quantify effectiveness, we performed intention-to-treat (ITT) (i.e., intervention effect estimated as per the village randomisation sequence for the intervention specified a priori in the trial protocol) and as-treated (AT) (i.e., intervention effect estimated as per the actual observed, in-the-field, village sequencing for the intervention; where a village may have been transitioned either before or after the week they were scheduled) analyses. In both ITT and AT analyses, outcome measurements during training periods are excluded from estimation.

Poisson linear mixed-effects modelling was performed on village-level observations to estimate the effect of the CIME intervention on malaria blood examination by RDT frequency (i.e., rate ratio). The intervention was modelled as a time-varying (monotonic) binary variable which indicated a village’s CHW intervention status as being either control (ICMV) or intervention (CIME) condition. Bootstrapped standard errors (n = 500 replications) were estimated in these models to provide correct inference in the face of possible response over-dispersion. A crossed-random effects (or non-nested) structure was used to correctly account for dependencies in testing (i.e., tests by the same cluster (village) were undertaken at different cross-sectional periods (weeks) and tests undertaken in the one period were from different villages), with random effects (i.e., intercepts) for both village (level-2) and week (level-2) crossed at the village test level (level-1). In addition to random effects for village and week (i.e., cross-sectional week clustering), the CIME intervention was modelled as a time-varying random effect at the village level to account for between-village differences in the nature of the effect (i.e., heterogeneity) of the intervention. The model also included fixed terms to adjust for the effect of time (linear week) and season (hot [March to April] and cool [November to February, reference group]) on blood examination frequency. The functional form of time was based on comparison of model fit across models featuring a range of polynomial degrees. The CIME intervention was modelled as a contemporaneous factor (i.e., having an immediate effect following training) for the primary analysis). Stata version 17 was used for all statistical analyses, sequence randomisation, and power estimation.

### Costs

For both CHW models, a program experience approach was used to estimate the total cost (one-off costs plus ongoing costs) in the three townships over the 24-weeks trial period. Expenditure reports were used to collate one-off and ongoing costs associated with each model in Microsoft Excel. Costs included one-off recruitment, procurement of medicines and commodities and training costs, plus ongoing costs of monitoring and supervision, support, assisted referral fees, recording and reporting, data collection and staff salaries.

All costs were calculated in United States Dollars (USD) and expenditure in Myanmar Kyats were converted into USD using the reference exchange rate of Central Bank of Myanmar (www.cbm.gov.mm) at the time of implementation (1US$ = 2100 Kyats). Human resources and infrastructures of the Ministry of Health at regional and township levels were not included in the calculation.

For both CHW models, we calculated and estimated (i) total cost per CHW per one-, three- and five-year period, (ii) total first year cost for each model for national rollout, assuming an indicative number of 20,000 CHWs nationwide regardless of geographical location, (iii) cost per RDT reported (total cost/number of RDTs) under each model. Finally, as the primary aim is to increase testing by RDT, the cost per additional RDT was calculated using the formula (additional cost of CIME model/additional RDTs in the CIME model).

### Role of the funding source

The study sponsors had no role in study design; in the collection, analysis, and interpretation of data; in the writing of the report; or in the decision to submit the paper for publication.

## Results

Originally, the study sought to include 140 CHWs servicing 140 villages across Ayeyarwady, Bago and Yangon Regions and Kayah State, Myanmar. But due to logistical constraints and disruptions relating to the COVID-19 pandemic and political instability, 72 villages in Hlegu, Kungyangon and Taikkyi Townships in Yangon Region were ultimately positioned to participate in the study. Of the original sample of 72 CHWs, several (n = 14) village CHWs randomised to study were either not contactable (n = 10) or chose not to participate after contact was made (n = 1 refused consent, n = 3 resigned as a CHW) and these CHWs were replaced with new CHWs randomised independently to intervention sequences, including an additional n = 2 CHWs recruited by township elders in villages that were previously thought not to be well-positioned to participate in the study—increasing the sample to n = 74 villages. As the study progressed, an additional n = 3 CHWs dropped-out (without formal contact and before attending training) with n = 1 of these departing CHWs replaced, bringing the total villages providing the intervention to n = 72 (Supplementary Material S6—Table S1).

In terms of the actual observed randomised sequence of villages between control and intervention conditions (which forms the basis of AT analyses and results), n = 10 villages were transitioned out of sequence. Of these, the majority (n = 8) were transitioned to the intervention after their randomised sequence. The largest discrepancy between the randomised and actual observed intervention sequence for a village or ICMV was 12 weeks (late).

Using an ITT framework, between Nov 1, 2021 and April 17, 2022, a total of 1656 weekly study measurements for RDT testing, 2886 malaria RDTs (control condition: 1365; intervention condition: 1521) were undertaken across the 72 villages during the 24-week study period (Fig. 2a, Table 1). Using an AT framework, a total of 2883 malaria RDTs were undertaken outside of training periods (control condition: 1381; intervention condition: 1502) (Fig. 2b). The mean (SD) malaria blood examination rate was 1.74 (2.50) tests per village per week and most tests (70.4%) were undertaken during the ‘cool’ season (Table 1). Three RDT-positive non-severe *P. vivax* cases (control: 2; intervention: 1; all male) were notified to basic health staff, treated as per the National Malaria Treatment Guidelines, and directly observed for malarial treatment compliance during the study.

**Table 1:** Malaria blood examination by rapid diagnostic test (RDT) and referral of suspected dengue, tuberculosis, diarrhoea and RDT-negative fever cases by control and intervention periods of the study.

Study characteristic	Control period	Intervention period	Total
Total no. of weekly measurements—RDT testing	852	804	1656
Season (n (%))			
Cool	390 (45.80)	776 (96.50)	1166 (70.40)
Hot	462 (54.20)	28 (3.50)	490 (29.60)
RDT tests per village/week (mean (SD))	1.70 (2.70)	1.79 (2.20)	1.74 (2.50)
RDT tests (total)	1365	1521	2886
Total no. of weekly measurements—referral	766	775	1541
Referrals per village/week (mean (SD))	0.11 (0.74)	0.28 (0.93)	0.20 (0.85)
Referrals (total)	86	217	303

From a total of 1541 weekly study measurements for referral, 303 referrals (control: 86; intervention: 217) were made across the 67 villages that participated for the entire 24-week study period. The mean (standard deviation) referral rate of suspected dengue, TB, diarrhoea or RDT-negative fever cases were 0.20 (0.85) referrals per village per week and majority of referrals (63%) were done during the ‘cool’ season (Table 1 and Supplementary Material S6—Table S2).

### Effectiveness of the co-designed expanded CHW (CIME) model

**Fig. 3:**
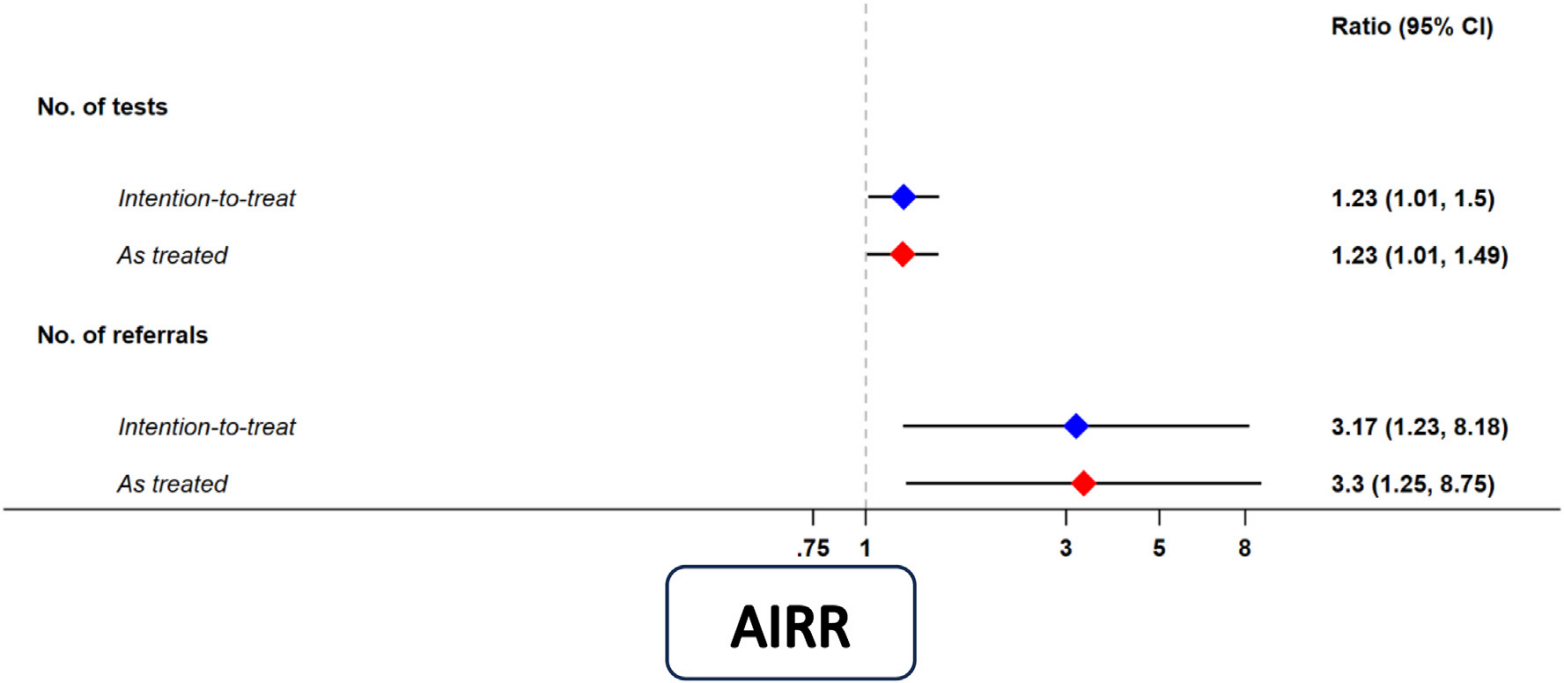
**Effectiveness of the CIME intervention compared to ICMV model on malaria blood examination by RDT and referral for suspected dengue, tuberculosis (TB), diarrhoea and RDT-negative fever.***Intention-to-treat* = intervention effect estimated as per the village block randomisation specified in the trial protocol. *As-treated* = intervention effect estimated as per the actual observed village block sequencing. AIRR—Adjusted incidence rate ratio; CIME—Community-delivered Integrated Malaria Elimination; ICMV—Integrated Community Malaria Volunteers.

**Fig. 4:**
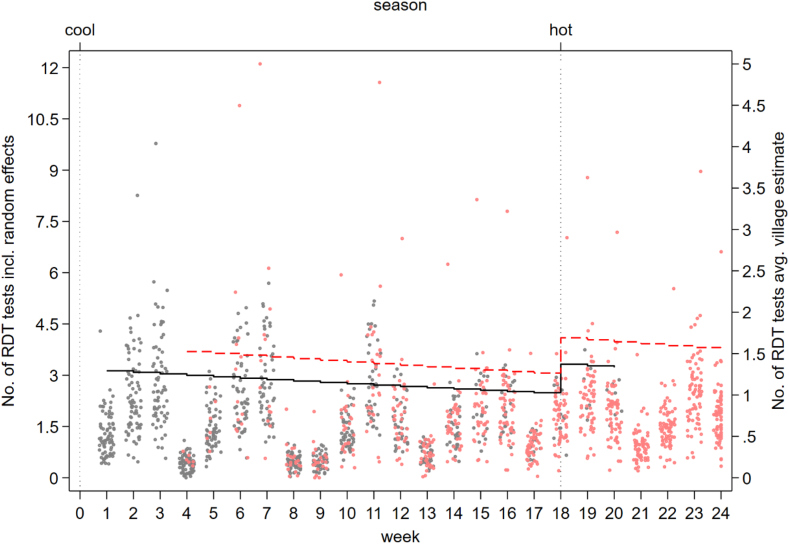
**Village malaria blood examination by RDT (random effects and village average) by ICMV and the CIME volunteers adjusted for time and season.** Black dots—Random effects, Black line—Villages average malaria blood examination by RDT (ICMV). Red dots–Random effects, Red dotted line–Villages average malaria blood examination by RDT (CIME). ICMV—Integrated Community Malaria Volunteer in control phase, CIME—Community-delivered Integrated Malaria Elimination community health worker in intervention phase. The dots represent the estimated values from the random effects for each value and the lines are the village average.

In ITT analyses, we observed a 23% relative increase in village RDT testing with the introduction of the CIME model (adjusted incidence rate ratio [AIRR] = 1.23, 95%CI = 1.01, 1.50, p = 0.036; control condition: adjusted (per week) incidence rate [AIR] = 1.75, 95%CI = 1.33,2.31; intervention condition: AIR = 2.16, 95%CI = 1.54,3.03), adjusting for time and season. The same effect of the intervention on village RDT testing was observed in AT analyses (AIRR = 1.23, 95%CI = 1.01, 1.49, p = 0.042; control condition: AIR = 1.74, 95%CI = 1.25,2.22; intervention condition: AIR = 2.13, 95%CI = 1.50,3.01) (Fig. 3 and Supplementary Material S6—Table S3). In both ITT and AT analyses, there was no change or secular trend in the rate of malaria blood examination per week, independent of the intervention and season (ITT: AIRR = 0.99, 95%CI = 0.97, 1.00, p = 0.071; AT: AIRR = 0.99, 95%CI = 0.97, 1.00, p = 0.090). In terms of malaria season, testing was approximately 36% higher in the ‘hot’ season and this effect was consistent across ITT and AT analyses (ITT: AIRR = 1.36, 95%CI = 1.15, 1.60, p < 0.001; AT: AIRR = 1.35, 95%CI = 1.14, 1.60, p = 0.001) (Fig. 4 and Supplementary Material S6—Table S3).

**Fig. 5:**
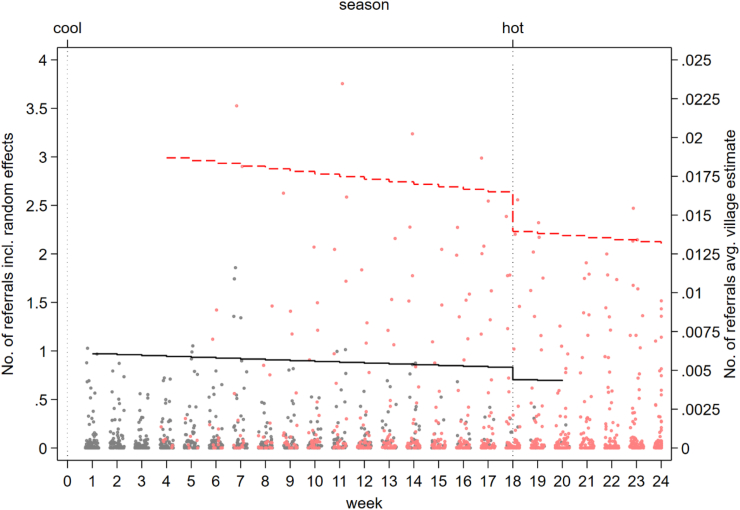
**Village referral of suspected dengue, tuberculosis, diarrhoea and malaria rapid diagnostic test-negative fever cases (random effects and village average) by ICMV and the CIME volunteers adjusted for time and season.** Black dots—Random effects, Black line—Villages average referral (ICMV). Red dots–Random effects, Red dotted line–Villages average referral (CIME). ICMV—Integrated Community Malaria Volunteer in control phase, CIME—Community-delivered Integrated Malaria Elimination community health worker in intervention phase. The dots represent the estimated values from the random effects for each value and the lines are the village average.

We observed a greater than 3-fold relative increase in village weekly referral of dengue, TB, diarrhoea or RDT-negative fever cases with the introduction of the CIME intervention in ITT (AIRR = 3.17, 95%CI = 1.23, 8.18, p = 0.017; control condition: AIR = .005, 95%CI = 0.000, 0.115; intervention condition: AIR = .016, 95%CI = 0.001, 0.336) and AT analyses (AIRR = 3.30, 95%CI = 1.25, 8.75, p = 0.016; control condition: AIR = .006, 95%CI = 0.000, 0.115; intervention condition: AIR = 0.019, 95%CI = .001, 0.366), adjusting for time and season (Fig. 3). In both ITT and AT analyses, there was no change/secular trend in the rate of referral per week, independent of the intervention and season (ITT: AIRR = 0.99, 95%CI = 0.92, 1.06, p = 0.793; AT: AIRR = 0.99, 95%CI = 0.92, 1.06, p = 0.716) (Fig. 5 and Supplementary Table S4).

### Cost-effectiveness of the expanded CHW model

Total cost of the CIME and ICMV models with 72 CHWs in three townships of Yangon Region over the trial period was US$ 50,776 ($ 3000 initial one-off + $ 47,776 ongoing operation) and $ 18,630 ($ 439 initial one-off + 18,191 ongoing operation), respectively. The total cost per CHW per one-, three- and five-year period was $ 2,992, $ 8893 and $ 14,794 respectively for the CIME model, and $ 1,168, $ 3492 and $ 5816 respectively for the ICMV model. Assuming that there are 20,000 villages in Myanmar, the total first year costs for the CIME and ICMV models were estimated to be $59,843,345 and $23,363,121, respectively. The cost per malaria RDT test reported over the trial period was $ 33 in the CIME model and $ 14 in the ICMV model (Table 2). Over the trial period the CIME model cost $ 206 per additional RDT conducted compared to the ICMV model.

**Table 2:** Estimated costs of the CIME and ICMV models for national rollout and costs per malaria blood examination by rapid diagnostic test (RDT) reported under each model.

Description	ICMV model (USD)	CIME model (USD)
Total one-off cost (for 72 volunteers)	438.70	3000.23
Total ongoing cost (for 72 volunteers)	18,190.76	47,775.36
Total cost (one-off + ongoing) for 72 volunteers	18,629.46	50,775.59
Cost per malaria RDT test reported	13.65	33.38
One-off cost per volunteer	6.09	41.67
Ongoing cost per volunteer per week	22.35	56.74
Total cost (one-off + ongoing) per volunteer for 1 year	1168.16	2992.17
Total cost (one-off + ongoing) per volunteer for 3 years	3492.28	8893.16
Total cost (one-off + ongoing) per volunteer for 5 years	5816.41	14,794.16
Total cost (one-off + ongoing) per year for 20,000 volunteers	23,363,121.07	59,843,344.76

CIME—Community-delivered Integrated Malaria Elimination; ICMV—Integrated Community Malaria Volunteers.

## Discussion

As malaria endemic countries, including those in GMS, progress towards malaria elimination and the malaria burden declines, so too does the blood examination rate and motivation of CHWs and end users. Sub-optimal malaria blood examination rates jeopardise the effectiveness of malaria elimination programs and the ability for countries to achieve WHO accreditation of malaria elimination. An expanded CHW model (malaria plus other primary health care services) has the potential to increase malaria blood examination rates and may have the greatest impact if co-designed with end users and beneficiaries. In a stepped-wedge cluster randomised controlled trial in Myanmar, we demonstrated that an expanded role of CHWs, providing services optimised by communities, CHWs and malaria key stakeholders (the CIME model) increased weekly malaria blood examination by RDT, as well as referral for other common infectious diseases, compared to the standard malaria CHW model (ICMV). While the CIME model was associated with additional costs for providing health services for common infectious diseases, these costs may be worth the investment as GMS countries move towards universal health care. Therefore, co-designing and expanding the role of CHWs in malaria endemic countries may be a highly effective and cost-effective public health strategy to maintain the annual blood examination rates required for malaria case detection, surveillance and WHO malaria elimination accreditation.

Highly effective CHW programs rely on delivering services based on community demand, but within the scope of the national health system. In this context, this study is the first to trial an expanded CHW model for malaria elimination settings co-designed by end users, beneficiaries and malaria stakeholders. By co-designing an expanded CHW model, the CIME model, which incorporated services for malaria elimination, dengue, TB, RDT-negative fever and diarrhoea, increased malaria blood examination rates by approximately one quarter. Furthermore, the expanded service package provided through the implementation of the CIME model was associated with a 3.3-fold relative increase in referrals for dengue, TB, childhood diarrhoea and RDT-negative febrile illness compared to the implementation of the current CHW model (ICMV). This demonstrates strong community uptake of services by end users and commitment by CHWs in the provision of malaria and expanded services although not all suspected cases were referred and not all referred cases actually visited health centres due to excessive reliance on the CHW’s treatment services by the community, long waiting time at the referral health centres and frequent unavailability of healthcare providers at the referred facilities. Findings from (unpublished) qualitative consultations with both community members and CHWs highlighted that the provision of symptomatic and assisted referral services for RDT-negative febrile cases may increase motivation and service uptake among end users. It resolves unsatisfactory end-of-the-service issues when an RDT is negative, a common experience previously reported from malaria-focused CHW models.^9^^,^^10^ The provision of services for childhood diarrhoea by CHWs was also viewed as a favourable addition. There is a strong evidence-base for the inclusion of interventions for childhood diarrhoea in malaria CHW models for reducing mortality in children under the age of five-years, with its inclusion in the integrated Community Case Management malaria CHW model which provides diagnosis and treatment of pneumonia, diarrhoea and malaria, and widely implemented across high-burden settings in Africa.^4^ This study shows that the inclusion of services for childhood diarrhoea can also contribute to the strong uptake of services and increase malaria blood examination rates in malaria elimination settings.

Across the GMS, CHWs provide essential malaria services and the findings of the implementation of the expanded CHW model may benefit other countries in the GMS and other regions experiencing declines in malaria blood examination rates by CHWs. Several countries in the GMS, aware of the potential of expanded CHW models for malaria elimination, are implementing or planning to implement an expanded CHW model to respond to the declining role and usefulness of malaria CHWs as well as to strengthen the primary healthcare system in the country. The expansion of CHW roles could extend beyond expanding the role of malaria CHWs to other CHWs (e.g., auxiliary midwives) to include malaria and other services for the malaria elimination phase. In Lao People’s Democratic Republic, the Ministry of Health plans to adopt the integrated Community Case Management model^26^ after a co-designing process and adapting it into the country context. This includes adding maternal and child health services into the CHW model guided by the local disease burden and evidence of health services demand in the community.^10^ Cambodia also plans to integrate additional interventions into an expanded CHW model (both village and mobile malaria CHWs) according to local epidemiology and demand, such as surveillance and health education for vector-borne diseases, health promotion and prevention for non-communicable diseases, and maternal and child health services.^12^ This integration is planned to occur phase by phase between 2021 and 2025.^12^ In Viet Nam and Thailand, malaria CHWs played an important role in both malaria control and elimination programs (although their role is limited to malaria prevention and referral of malaria RDT positive cases). Currently the malaria burden in Viet Nam is very low (441 cases in 2023).^27^ However, the role of CHW is at the final stages of the malaria elimination and prevention of malaria reintroduction phases, and maintaining high malaria blood examination rates will be critical. In this context, this trial provides evidence for the use of an expanded CHW model in malaria elimination settings, and impetus for the deployment of context-specific expanded CHW models in other GMS countries and regions that aim to maintain malaria blood examination rates by CHWs.

A strength of this study is that the intervention was co-designed by end-users, beneficiaries and malaria stakeholders. According to an unpublished mixed-methods nested study, the implementation of the CIME model was feasible and acceptable to community members, local and health stakeholders. Local ownership and buy-in to the implementation of the co-designed CHW model may also prove it to be more sustainable in the long run. Over the six-month course of this study, malaria blood examination rates by RDT were maintained. Previous observational studies have observed sustained blood examination rates for more than two years after the introduction of expanded CHW services into general healthcare during the malaria control phase.^11^^,^^15^ Adoption of an expanded CHW model in any region needs continued refinement to meet the changing needs of the implementers and end users as well as to address emerging infectious diseases such as COVID-19. Refinement of the model may also include emerging strategies or interventions for malaria or other priority diseases. For example, there is recent evidence for the role of CHWs in the deployment of quantitative Glucose-6-Phosphate-Dehydrogenase testing for safe administration of high dose primaquine radical cure for *P. vivax*,^28^^,^^29^ which would advance the current CIME model whereby CHWs were assigned to ensure radical cure of *P. vivax* cases with 14-day low dose primaquine along with monitoring of adverse events.

Originally, this study planned to implement the expanded CHW (CIME model) in 140 CHW servicing villages across four states and regions with different rates of malaria transmission, culture, terrain and transportation, and health system capacity in Myanmar. However, due to the COVID-19 pandemic and an ongoing political crisis, it was only possible to implement in 72 CHW servicing villages in Yangon Region; a region with the strongest health system capacity and one of the lowest malaria transmission rates in Myanmar. This meant the study was unable to quantify the impact of the CIME model on malaria case detection rates (only three malaria positive RDTs over the trial duration) and that the findings of this study may not be generalisable with respect to other states and regions which exhibit varying malaria transmission rates, terrain structures and health system capacities. Given the observed level of impact of the CIME intervention on testing rates (23% relative increase; minimum detectable difference estimated in a priori power estimation was 13%), the smaller than targeted sample size in our study (n = 140 reduce to n = 72 villages), although reducing notional power and possibly precision (all things being equal), our study was nonetheless able to observe statistically significant differences. In addition, in Myanmar the current CHW model (ICMV) already provided (albeit reduced) services for dengue and tuberculosis (in addition to low priority/low burden diseases of these communities: HIV/AIDS, leprosy, lymphatic filariasis).^8^^,^^9^^,^^13^ Therefore, the magnitude of effect of the CIME intervention on malaria blood examination rates may be greater if compared to a CHW model which provides malaria services alone.

Basic essential health services could be provided by CHWs effectively and affordably which may facilitate the achievement of universal health coverage if the CIME model is scaled-up nationally. The CIME model costed $206 per additional RDT performed compared to the ICMV model, however the additional RDTs conducted are only one element of the benefits of the CIME model over the ICMV model. The CIME model also increased referrals and therefore likely increased access to treatments for dengue, TB, diarrhoea and RDT-negative febrile illnesses that are not included in our cost analyses. There are also cost benefits which are not captured. The increased uptake of malaria RDT testing will improve surveillance and lead to faster case detection and notification, reducing the risk of onward transmission and subsequent malaria cases.^3^ Prompt referral and treatment of other infectious diseases captured in this expanded CHW model will also reduce the likelihood of onward transmission which will have significant positive health and economic impacts. Delivering quality primary healthcare services to the community through a strong CHW programme could also provide important surveillance for other infectious diseases approaching elimination and could contribute to the goal of universal health coverage.^30^

In summary, the expanded co-designed CHW (CIME) model is effective in increasing malaria blood examination by RDT in the context of declining malaria cases, and therefore presents as a useful intervention for malaria elimination and prevention of re-introduction programs which at their core need to maintain high rates of malaria blood examination. Although an expanded CHW model costs more than the current CHW model, it could form part of primary health care programs and a pathway to universal health coverage. The co-designed CIME model could be field implemented or integrated in the evolution of CHW models in other malaria endemic countries who rely on a network of CHWs to deliver malaria services particularly other GMS countries given the similar malaria epidemiology and goal of malaria elimination.

## Contributors

Win Han Oo, Paul A. Agius and Freya J.I. Fowkes designed the study. Win Htike, Pwint Phyu Phyu, May Chan Oo, Ei Phyu Htwe, Aung Khine Zaw, Kaung Myat Thu and Naw Hkawng Galau led field implementation and data collection under the supervision of Tun Min and Nay Yi Yi Linn. Paul A. Agius, Win Han Oo, Win Htike, May Chan Oo, Ei Phyu Htwe, Aung Khine Zaw, Kaung Myat Thu and Nick Scott cleaned and analysed the datasets. Paul A. Agius and Nick Scott supervised statistical and cost analyses respectively. Win Han Oo wrote the manuscript under the supervision of Freya J.I. Fowkes and Paul A. Agius. Kyawt Mon Win, Nay Yi Yi Linn, Tun Min, Julia C. Cutts, Julie A. Simpson and Katherine O’Flaherty contributed to manuscript drafting. All authors reviewed the manuscript, provided critical inputs and approved the final manuscript.

## Data sharing statement

The deidentified datasets generated and analysed will be made available to others with publication upon reasonable request to corresponding authors and contingent on the approval of Myanmar Ministry of Health. The study protocol with data analysis plan is available as Supplementary Material S1.

## Editor note

The Lancet Group takes a neutral position with respect to territorial claims in published maps and institutional affiliations. As Burmese names do not have family names, full names of all authors have been used in certain sections (instead of author initials).

## Declaration of interests

The authors declare that they have no competing interests.
