# Supplementary material for: Effectiveness of an expanded role for community health workers on malaria blood examination rates in malaria elimination settings in Myanmar: an open stepped-wedge, cluster-randomised controlled trial
Source: Lancet Reg Health Southeast Asia. 2024 Oct 17;31:100499. doi: 10.1016/j.lansea.2024.100499 (PMC11531616; doi:10.1016/j.lansea.2024.100499)
Supplement: Supplementary Material S1 [file mmc1.docx]

Published in *BMJ Open* <https://bmjopen.bmj.com/content/11/8/e050400>

**Evaluation of the effectiveness and cost-effectiveness of a** **Community-delivered Integrated Malaria Elimination (CIME) model in Myanmar: Protocol for an open stepped-wedge cluster-randomised controlled trial**

Win Han Oo^1*^, Aung Thi^2^, Win Htike^1^, Paul A. Agius^1,3^, Julia C. Cutts^1,4^, Kyawt Mon Win^2^, Nay Yi Yi Linn^2^, Wint Phyo Than^2^, Galau Naw Hkawng^1^, Kaung Myat Thu^1^, May Chan Oo^1^, Katherine O’Flaherty^1^, Ellen Kearney^1^, Nick Scott^1^, Pwint Phyu Phyu^2^, Aung Thu Htet^2^, Ohnmar Myint^2^, Lwin Lwin Yee^2^, Zay Phyo Thant^2^, Aung Mon^2^, Soe Htike^2^, Thet Pan Hnin^2^, Freya J.I. Fowkes^1,3,5*^

^1^. Disease Elimination Program, Burnet Institute, VIC, Australia

^2^. Department of Public Health, Myanmar Ministry of Health and Sports, Myanmar

^3^. Department of Epidemiology and Preventive Medicine, Monash University, VIC, Australia

^4.^ Department of Medicine at the Doherty Institute, University of Melbourne, Melbourne, Australia

^5^. Melbourne School of Population and Global Health, University of Melbourne, VIC, Australia

*Corresponding authors

Win Han Oo: [winhan.oo@burnet.edu.au](mailto:winhan.oo@burnet.edu.au) and Freya Fowkes: [Freya.fowkes@burnet.edu.au](mailto:Freya.fowkes@burnet.edu.au), 85 Commercial Road, 3004, VIC, Australia.

Word count – 3769 (excluding cover page, abstract, declarations, references, figure legends and list of supplemental materials)

# **Abstract**

## Introduction

In the Greater Mekong Subregion, community health workers, known as malaria volunteers, have played a key role in reducing malaria in the control phase, providing essential malaria services in areas with limited formal health services. However, the motivation and social role of malaria volunteers, and malaria testing rates, have declined with decreasing malaria burden and reorientation of malaria programs from control to elimination. Provision of additional interventions for common health concerns could help sustain the effectiveness of volunteers and maintain malaria testing rates required for malaria elimination accreditation by the WHO.

## Methods and analysis

The Community-delivered Integrated Malaria Elimination (CIME) volunteer model, integrating interventions for malaria, dengue, tuberculosis, childhood diarrhoea and malaria Rapid Diagnostic Test (RDT)-negative fever, was developed based on global evidence and extensive stakeholder consultations. An open stepped-wedge cluster-randomised controlled trial, randomised at the volunteer level, will be conducted over 6 months to evaluate the effectiveness of the CIME model in Myanmar. One hundred and forty Integrated Community Malaria Volunteers (ICMVs, current model of care) providing malaria services in 140 villages will be retrained as CIME volunteers (intervention). These 140 ICMVs/ villages will be grouped into 10 blocks of 14 villages, with blocks transitioned from control (ICMV) to intervention states (CIME), fortnightly, in random order, following a 1- week training and transition period. The primary outcome of the trial is blood examination rate determined by the number of malaria RDTs performed weekly. Difference in rates will be estimated across village intervention and control states using a generalised linear mixed modelling analytical approach with maximum likelihood estimation.

## Ethics and dissemination

The study was approved by Institutional Review Board, Myanmar Department of Medical Research (Ethics/DMR/2020/111) and Alfred Hospital Ethics Review Committee, Australia (241/20). Findings will be disseminated in peer-review journals, conferences and regional, national and local stakeholder meetings.

*Trial registration number:* NCT04695886

*Key words*: volunteer, community-delivered model, malaria elimination, primary health care, Myanmar

# **Article Summary**

## Strengths and limitations of this study

- This study will provide the first quantitative evidence, using a rigorous design and methodology, to evaluate whether malaria volunteers delivering expanded interventions for common health concerns (CIME model) can increase the effectiveness of malaria volunteers in malaria elimination programmes
- The intervention was developed in collaboration with the Myanmar National Malaria Control Program and the study will measure implementation research outcomes such as effectiveness of the intervention measured by malaria blood testing rate, cost-effectiveness, acceptability, feasibility and fidelity. Therefore, the CIME model can be easily transferred or adapted to national guidelines of Greater Mekong Subregion countries in order to meet national and regional malaria elimination targets
- The strength of the stepped-wedge cluster-randomised controlled trial design is that it will allow investigation of any temporal effects of the intervention both between and within villages and, given certain conditions and appropriate statistical modelling, can offer superior statistical power as it models cluster level error in the outcome more efficiently.
- The complex sampling strategy will yield a representative trial sample given a set of known population parameters, and this will provide a greater level of external validity to the findings of the trial; and will be a major strength of the research in the context of the intention-to-treat nature of the study.
- However, the primary limitation of the stepped-wedge cluster-randomised controlled trial design is that it can induce confounding between the effects of calendar time and intervention exposure which will need to be appropriately adjusted for in all statistical analyses.

**Introduction**

Since 2000, global malaria-attributable morbidity and mortality has declined significantly, in particular in the South-East Asia region where malaria cases and death have reduced by >70%.^1^ However, emerging resistance to the first-line treatment for malaria, the artemisinin derivatives, in the Greater Mekong Sub-region (GMS) threatens progress towards regional, and global, malaria milestones.^2-4^ To end the malaria epidemic and to stop the spread of resistance in, and from, the GMS, the global conceptual framework of malaria has changed in recent years from control to elimination, with the WHO and Asia-Pacific leaders having committed to eliminate malaria from the region by 2030.^5 6^

In the GMS, community health workers, known as malaria volunteers, have played a key role in reducing the malaria burden in the malaria control phase, providing essential malaria services in rural areas where the coverage of formal health services is limited.^7^ However, the community-delivered models that have worked well for malaria control may not work well for malaria elimination. In Myanmar, and many other GMS countries, in parallel with the switch from interventions for malaria control to elimination, the motivation and social role of malaria volunteers has dropped along with the declining malaria burden.^8^ As the proportion of febrile illnesses attributable to malaria declines, malaria volunteers are less likely to be able to treat a case of febrile illness, and their perceived utility in the community is compromised.^9^ As a result, malaria testing rates have declined in many areas,^9^ threatening progress towards achieving Myanmar’s National Plan for Malaria Elimination^10^ recommendation of an optimal annual blood examination rate of 10% and above for populations at risk (the annual blood examination rate in 2016-2018 was 6.1%^11^). Blood examination rate targets are designed to accurately quantify the annual parasite incidence, a critical indicator to activate the malaria elimination program in Myanmar and ultimately for achieving WHO certification of malaria elimination.

Provision of an expanded range of volunteer-delivered services to the community is a potential strategy to provide more opportunities for malaria volunteers to test for malaria and to maintain the annual blood examination rates at the recommended level. This rationale is supported by studies that demonstrate that additional care packages can increase the utilisation of malaria volunteers. For example, the Integrated Community Case Management programmes in which malaria volunteers deliver case management for malaria, pneumonia, diarrhoea, as well as malnutrition screening and nutrition counselling to children under five by, has demonstrated good acceptability and effectiveness in reducing under five mortality and malaria burden in Africa,^12-16^ as well as in Myanmar.^17^ Similarly, in a recent observational study in Myanmar, malaria volunteers providing a basic health care package (that included management for respiratory tract infections, diarrhoea and skin infections, detection and treatment of acute malnutrition, active case detection and referral of tuberculosis (TB) and referral for other severe illnesses) saw an increase in the volunteers’ blood examination rate using malaria RDTs ^9^.

Recognising the challenge of sustaining malaria testing in an elimination setting, the Myanmar National Malaria Control Program (NMCP) commenced a national roll out of the Integrated Community Malaria Volunteer (ICMV) model in 2017-18.^18^ This program expanded the scope of malaria volunteers to include additional screening and referral services for other diseases including dengue, lymphatic filariasis, TB, HIV/AIDS and leprosy (detailed description in **Supplemental material 1**).^19^ However, introduction of the ICMV model did not result in a significant improvement in the annual blood examination rate which plateaued at around 6% between 2016 and 2018.^11^ While the rationale behind service integration in the ICMV programme is strong, the choice of additional services offered by the ICMVs could be improved by incorporating the perspectives of the end users (community members/leaders and current volunteers) and malaria stakeholders (NMCP and other malaria implementing partners). Improvement of the current malaria volunteer model may increase rates of malaria testing in the elimination phase. To address this, in collaboration with the NMCP, we undertook a global literature review and qualitative research study engaging key community and malaria stakeholders in Myanmar to explore perspectives and identify strategic and operational barriers and enablers of malaria elimination in community-delivered models.^7 20 21^ Based on the evidence from these primary stages, and in further collaboration with the NMCP, we designed an optimal intervention package for malaria volunteers to deliver in the elimination phase known as ‘The Community-delivered Integrated Malaria Elimination model (The CIME model)’. This model is specific to the GMS context of elimination and offers services for malaria elimination, dengue, TB, non-malaria fever and childhood diarrhoea, whereas some interventions previously included in the ICMV model (for lymphatic filariasis, HIV and leprosy) have been removed. It is designed to execute all essential community-based interventions of the malaria elimination programme, and maintain high rates of testing for malaria in the community by providing treatment and prevention for other key diseases. If proven to be effective and endorsed by the Myanmar Ministry of Health and Sports, implementation of the CIME model could contribute to achieving the national malaria elimination goal of 2030 by maintaining high annual blood examination rates and ultimately fulfilling the requirements of malaria elimination certification by WHO.

This paper presents the protocol of a stepped-wedge cluster-randomised controlled trial which aims to estimate the CIME model’s effectiveness in increasing malaria testing rates, and access to malaria and other health services, as well as its acceptability, feasibility and cost-effectiveness compared with current community-delivered models in Myanmar (ICMV model) in the context of the malaria elimination phase.

# **Methods and analysis**

## Intervention

The CIME model intervention for malaria testing, treatment and referral integrates services for malaria, dengue, TB, childhood diarrhoea and RDT-negative fever (**figure 1**, **table 1** and online supplemental material 2). Any concomitant care received by community members will not preclude inclusion in the study.

Table 1: Interventions for each integrated disease in the CIME volunteer model

| **Diseases** | **Interventions** |
| --- | --- |
| Malaria | Interventions in the ICMV Model to be continued in the CIME Model   - Malaria diagnosis using RDT (SD BIOLINE Malaria Ag P.f / Pv) - Treatment and/or assisted referral of malaria cases according to the national malaria treatment guidelines of Myanmar - Prevention interventions   - Behavioral change communication activities,   - Assistance in distribution of long-lasting insecticidal nets and mosquito repellent - Community mobilisation for malaria elimination activities - Contribution to fieldwork for malaria microstratification by malaria transmission intensity, and in surveillance and research activities - Reporting of malaria cases within 24 hours of diagnosis using direct phone call and/or SMS text message to their immediate supervisor/ local vector borne disease control program staff   Serving as directly observed treatment providers |
|  | New intervention to be added   - Conducting preliminary case investigations and differentiating indigenous and imported cases - Assisting in formal case and foci investigation, and response teams in community mobilization, information collection and larval source management activities - Serving as translators for formal case and foci investigation teams in areas where people use languages other than Burmese |
| Malaria RDT-negative fever | New intervention to be added   - Prevention and health education - Symptomatic treatment with antipyretics and immediate assisted referral |
| Dengue | Interventions in the ICMV Model to be continued in the CIME Model   - Assisting vector borne disease control program staff and basic health staff in dengue prevention and vector control activities - Referral of suspected dengue cases to the nearest health center - Serving as a role model for dengue prevention and vector control activities in the community using a positive deviance approach |
| Tuberculosis | Interventions in the ICMV Model to be continued in the CIME Model   - Detection and referral of suspected TB patients by checking TB signs and symptoms - Contact tracing of TB patients in their community - Serving as directly observed treatment providers - Following up TB patients lost-to-follow-up (defaulter tracing) - Helping TB patients in follow-up sputum examinations - Assisting basic health staff in TB health education talks and active case detection activities |
| Childhood diarrhea | New intervention to be added   - Health education and water sanitation and hand hygiene promotion - Diagnosis - Clinical diagnosis and dehydration assessment - Treatment and referral - Rehydration therapy using oral rehydration solution and oral Zinc tablet - Assisted referral to health centers |

## Outcomes

Data required to determine the primary outcome of number of blood examinations for malaria (outcome 1, **table 2**) and secondary outcomes 2A-2B, 2D-2K (**table 2**) will be recorded by volunteers in a paper-based CIME volunteer record book which records details of each client visit to the volunteer at the time of consultation. RDT cassettes will be kept and stored as per standard operating procedures (online supplemental material 3**)** where possible to extract DNA and antibodies to determine secondary molecular and serological outcomes 2C and 2L-2N (**table 2**). At the end of the intervention trial, a mixed-methods study will be conducted (online supplemental material 4) to collect data on secondary outcomes 2O-2R. This will include a cross-sectional survey of community members in selected villages to explore the acceptability of the CIME model and feasibility of implementing the CIME model in their community. A qualitative study involving semi-structured interviews for key health stakeholders and community leaders and focus group discussion (FGD) with CIME volunteers will be conducted. A qualitative study involving observational fieldwork of CIME volunteers (2-4 ICMVs per township) will be conducted to observe their daily activities. Finally, a cost-analysis of the CIME model will be undertaken **(table 2)**.

| **Table 2: Primary and Secondary outcome measures of the study** | | | |
| --- | --- | --- | --- |
| ***Outcome Measures*** | | ***Measurement method*** | ***Time frame*** |
| ***Primary outcome:*** | |  |  |
| **1** | 1. Number of blood examinations for malaria | RDT (SD BIOLINE Malaria Ag *P.f / P.v*)* | Weekly |
| ***Secondary outcomes:*** | |  |  |
| **2A** | *Plasmodium* spp. infection detected by RDT | RDT* | Weekly |
| **2B** | *Plasmodium* spp. infection reported to volunteers immediate supervisors within 24 hours | RDT* | Weekly |
| **2C** | *Plasmodium* spp. infection detected by PCR | PCR | Weekly |
| **2D** | Larval source management | Activity recording* | Weekly |
| **2E** | Suspected dengue cases referred | Clinical assessment* | Weekly |
| **2F** | Suspected tuberculosis (TB) cases referred | Clinical assessment* | Weekly |
| **2G** | Tuberculosis: Directly observed Treatment Short Couse | Activity recording* | Weekly |
| **2H** | Diarrheal cases diagnosed, treated and referred | Clinical assessment* | Weekly |
| **2I** | RDT-negative fever cases | Thermometer and RDT* | Weekly |
| **2J** | Patients with confirmed malaria who received first-line antimalarial treat | Treatment with ACT or CQ and PQ* | Weekly |
| **2K** | Data accuracy and completeness in reporting of malaria cases | Malaria cases recorded by volunteers | Weekly |
| **2L** | Malaria drug resistance-associated mutations | PCR, sequencing | Weekly |
| **2M** | Seroprevalence of malaria-associated antibodies | ELISA | Weekly |
| **2N** | Levels of malaria-associated antibodies | ELISA | Weekly |
| **2O** | Acceptability of the CIME model by villagers | Self-report: Cross-sectional survey | At 6 months |
| **2P** | Acceptability of the CIME model by CIME volunteers | FGD and fieldwork observations | At 6 months |
| **2Q** | Acceptability of the CIME model by stakeholders | Semi-structured interview | At 6 months |
| **2R** | Cost-effectiveness of the CIME model | Cost data from CIME volunteers and NMCP consultations | At 6 months |
| An extended version of this table can be found at Clinicaltrials.gov (registration number: NCT04695886).  *Undertaken and recorded by volunteers.  ACT, artemisinin-based combination therapy; CIME, Community-delivered Integrated Malaria Elimination; CQ, chloroquine; FGD, focus group discussions; PQ, primaquine; RDT, rapid diagnostic test. | | | |

## Study setting and population

Villages in townships of Ayeyarwaddy, Bago and Yangon Regions and Kayah State in Myanmar will be included in the CIME model trial (**figure 2**). These states and regions are low malaria transmission areas and annual parasite incidence in almost all townships is less than 5 in 2019. The malaria case load is higher in rainy reason (May/June to October/November) in these areas. The study participants will include ICMVs who will be re-trained as CIME volunteers during the course of the study and the community members in the service catchment areas of those volunteers.

## Patient and Public involvement

Community members at risk of malaria were not involved in setting the research question or the outcome measures, but they participated in community consultations, the findings of which contributed to the development of the CIME intervention.

## Eligibility criteria

Townships and NMCP volunteer occupied villages within the township will be screened against the following exclusion criteria by investigators and NMCP staff**.**

A township will be excluded from the study if it:

- does not have an NMCP provided ICMV network
- has ongoing armed conflict
- does not have vector borne disease control program staff or malaria focal person
- its location is not geographically or politically feasible for NMCP staff from the State/Regional capital city to conduct regular supervision visits

A village will be excluded from the study if it:

- has an annual parasite incidence ≥5.00 per 1000 population in 2019
- is too remote to facilitate execution of the CIME model completely
- has a government public health facility
- has no mobile network coverage
- is in an ongoing armed conflict zone, or
- has an ICMV program operated by any organizations other than NMCP

## Study design

An open stepped-wedge cluster-randomised controlled trial,^22^ randomised at the volunteer level (i.e. the volunteer and the village/workplaces they service) (**figure 3**), will be conducted over a 6-month period to evaluate the effectiveness and cost-effectiveness of the CIME model. In this approach, the CIME model intervention will be implemented sequentially in a minimum of 140 villages serviced by 140 CIME volunteers. All the community members in sampled villages will receive the CIME model intervention, but the timing of the intervention implementation will be determined at random using a computerised random allocation sequence with de-identified village data. The randomisation process will be performed using randomisation code written by the trial statistician. Villages will be randomly allocated, independent of geographical location, to 10 ordered blocks of 14 villages, with blocks transitioned from control (ICMV) to intervention (CIME) states at fortnightly intervals following a 1-week training and transition period, beginning in July 2021 (the October/November peak in malaria transmission during the 6-month study period). This follows an initial 2-week baseline control period at the start of the study where all village sites will use the ICMV model exclusively (f**igure 3**). The primary outcome, number of blood examinations by RDT undertaken, will be measured weekly. All ICMV volunteers in the villages of the same block will be trained as CIME volunteers at the same time (in the same week) and will subsequently transition to CIME volunteers (the intervention state). Training periods will not contribute data to the trial.

## ICMV recruitment, training, and supervision

Existing volunteers in the NMCP ICMV network will be informed that they have been selected to participate in the CIME trial at least 1month prior to the commencement of the study in their villages. Volunteers will be provided with a Participant Information and Consent Form (online supplemental material 5) before providing written informed consent to participate in the study. Every enrolled CIME volunteer will receive initial malaria elimination training and induction training for the other four diseases (dengue, TB, childhood diarrhoea and RDT-negative fever). The CIME volunteer will be trained in malaria case investigation methodology and tools to conduct initial investigation when RDT positive patient(s) are detected (a more detailed description of volunteer training, management and support is provided in online supplemental material 2). During the 6-month study period NMCP staff, research staff, or where appropriate basic health staff, will conduct at least bimonthly monitoring visits to villages and employ rigorous data quality assurance systems (online supplemental material 2).

## Sampling

A total of 140 villages will be sampled using a two-stage sampling process. At the first stage, two townships from each of the four included states/regions (Ayeyarwaddy, Yangon, Bago, Kayah) will be sampled randomly with probability proportional to the size of the township. At the second stage, a fixed number of 18 villages will be sampled at random from each selected township to provide equal selection probabilities for villages. The number of villages sampled in each township is inflated for an expected village-level non-response of approximately 3%.

## Power

Assuming 140 villages, a baseline malaria RDT testing rate of approximately 2 tests per week, an estimate of between-village heterogeneity in testing (ICC) of *ρ*=0.30 (estimated from analyses of unpublished trial data^23^), the study will be powered (90%) to detect a minimum 13% relative increase (rate ratio=1.13) in testing attributable to the intervention (α=5%). Power estimation was explicitly based on estimation of an intervention effect using trial data from a stepped-wedge cluster randomised controlled trial design assuming analysis by generalised linear mixed modelling.^24^

## Data analysis

*Statistical analysis plan*

The difference in rates of the primary outcome, blood examination rate as measured by the number of RDTs conducted by volunteers, will be estimated across intervention and control states (intention-to-treat, given the study is estimating effectiveness) using generalised linear (eg, Poisson or negative-binomial link functions) mixed modelling with maximum likelihood estimation. Given the dependencies in the longitudinal data, village and time will be modelled as crossed random effects and study group as a time-varying monotonic factor adjusted for both time and seasonality. Heterogeneity in the effect of the intervention will be explored through estimation of a village-specific random effect in extended modelling. Temporal and spatial trends of RDT testing will also be explored including analysis of the effectiveness of the intervention across the study period. Additional, perprotocol type analyses involving intervention by effect moderator interactions will also be undertaken to estimate the extent to which different levels of intervention fidelity (assessed by a range of factors relating to village level acceptability and performance of the application of the intervention) impact RDT testing. Analyses of secondary outcomes and population demographics will involve a similar methodological approach, with link functions and response distributions specific to outcome type. In both descriptive and primary outcome trial analyses, sampling weights will be derived and applied in analyses where village selection probabilities are not equal—possible if the achieved number of villages sampled across townships varies. Furthermore, cluster robust standard errors will be used in all descriptive analyses given the complex sampling design. Data analysis for the stepped-wedged cluster randomised controlled trial will be performed using STATA V.15.

*Cost analysis*

A cost analysis will be conducted to estimate the average cost per annum of the CIME model (including implementation costs in the first year) and of the standard models. Economic costs will be estimated from a health systems perspective, for screening, treatment and referral services provided by the CIME volunteers (eg, excluding secondary costs for services they are referred to). A programme experience approach will be used, supplemented by consultations with the NMCP to capture any costs that are missed (eg, due to integration / reliance on existing services). Itemised costs from the study will be available and classified as implementation related or ongoing. The resulting cost outcomes will be used to estimate the cost per additional RDT resulting from the intervention, after 1year and by 2030.

# **Ethics and dissemination**

## Protocol and registration

This study protocol (version 1.0 dated 22 September 2020) adheres to the SPIRIT (Standard Protocol Items: Recommendations for Interventional Trials) statement (Supplemental material 6). The trial was prospectively registered on clinicalTrials.gov (NCT04695886) on 3 January 2021 and Myanmar Health Research Registry (PLRID-00948_V1) on 11 November 2020.

## Ethics statement

The study was approved by Institutional Review Board, Myanmar Department of Medical Research, approval number (Ethics/DMR/2020/111) and Alfred Ethics Review Committee, Victoria, Australia (241/20).

## Informed consent

Written consent will be obtained by researchers from ICMV participants before data collection and implementation of CIME model. As community members will continue receiving health services from the volunteer, either ICMV or CIME, no written consent will be obtained from the community members who receive services. The written consent forms and consent waiver request (Supplemental material 5) have been submitted and approved by Institutional Review Board, Myanmar Department of Medical Research to waive the need for an informed consent with regards to the collection, storage and further laboratory analysis of the RDT cassettes that would otherwise be discarded after use by study volunteers.

If an ICMV participant withdraws from the study during implementation, data collection pertaining to that person will stop but data participants provided before withdrawal from the study will be included in the analysis. If a volunteer withdraws, they will return to practicing as an ICMV volunteer and will continue reporting to the Myanmar NMCP as per the requirement of NMCP.

Data custodians will be the principal investigators and trial statistician at the Burnet Institute, Melbourne and Myanmar offices.

## Dissemination

A final technical report will be produced and a final dissemination workshop will be held with the NMCP, Myanmar Department of Medical Research and other malaria implementing partners to disseminate the findings of the study. The malaria related study findings will be provided to NMCP and secondary outcomes will be shared with respective departments/programmes in Myanmar Ministry of Health and Sports. Findings may also be presented at international and national conferences and published in peer-reviewed journals.

# **Declarations**

## Competing interests

The authors declare that they have no competing interests.

## Funding

This study was funded by an operational research grant (2018–21) from The Global Fund through United Nations Office for Project Services (Grant Number: 20864-003-38), with Dr Win Han Oo and Professor Freya Fowkes as principal investigators. Additional funding support was also received from the Australian National Health and Medical Research Council (Career Development Fellowship (#1166753) and Australian Centre of Research Excellence in Malaria Elimination to FJIF (#1134989). The Burnet Institute is funded by a Victorian State Government Operational Infrastructure Support grant. Investigators from Myanmar NMCP are government staff and their salaries and infrastructure are contributed by Myanmar Ministry of Health and Sports. The funders have no input on the design of the study, collection, analysis, interpretation and publication of the study results

## Contributors

WHO and FJIF conceived the study and with PA, WH and JCC designed the trial with inputs from all authors. PA conceived statistical analysis and NS specified the cost analysis. KO and EK contributed to laboratory aspects of the protocol. WHO, GNH, MCO, AT, WPT, KMW, KMT, AM, ATH, NYYL, OM, LLY, ZPT, AM, SH, TPH and PPP assisted in the logistics of setting up study sites. MCO led the Alfred Ethics applications. GNH and WH led the protocol submission to Institutional Review Board, Myanmar Department of Medical Research which was defended by WHO and AT. The paper draws from the trial protocol which was written collectively, under the leadership of WHO and FJIF. All authors reviewed and approved the final manuscript.

## Acknowledgements

Colleagues from Myanmar Ministry of Health and Sports: Dr Thandar Lwin, Dr Than Naing Soe, Dr Aye Mon Mon Kyaw, Dr Myat Min Tun and other NMCP staff at central, state/regional and township levels are acknowledged for their administrative and technical support that resulted in approval of the study. Colleagues from the Burnet Institute including Mr Chad Hughes, Ms Julie Tartaggia and Dr Phone Myint Win are acknowledged for their peer and logistic support.

# References

1. World Health Organization. World Malaria Report 2020: 20 years of global progress and challenges. In: Organization WH, ed. World Malaria Report. Geneva: World Health Organization, 2020.

2. Imwong M, Dhorda M, Myo Tun K, et al. Molecular epidemiology of resistance to antimalarial drugs in the Greater Mekong subregion: an observational study. *The Lancet Infectious Diseases* 2020 doi: 10.1016/S1473-3099(20)30228-0

3. Dondorp AM, Nosten F, Yi P, et al. Artemisinin resistance in Plasmodium falciparum malaria. *The New England journal of medicine* 2009;361(5):455-67. doi: 10.1056/NEJMoa0808859 [published Online First: 2009/07/31]

4. Hamilton WL, Amato R, van der Pluijm RW, et al. Evolution and expansion of multidrug-resistant malaria in southeast Asia: a genomic epidemiology study. *The Lancet Infectious Diseases* 2019;19(9):943-51. doi: 10.1016/S1473-3099(19)30392-5

5. World Health Organization. Meeting of the Malaria Policy Advisory Committee. Summary report. Geneva, Switzerland: World Health Organization, 2015:4.

6. World Health Organization. Countries of the Greater Mekong are stepping up to end malaria. *WHO’s Mekong Malaria Elimination Programme* 2018 November 2018; WHO’s Mekong Malaria Elimination Programme(November 2018). <https://apps.who.int/iris/bitstream/handle/10665/276213/WHO-CDS-GMP-MME-2018.03-eng.pdf> (accessed 10.05.2019).

7. Win Han O, Hoban E, Gold L, et al. Community demand for comprehensive primary health care from malaria volunteers in South-East Myanmar: a qualitative study. *Malar J* 2021;20(1):19. doi: 10.1186/s12936-020-03555-4 [published Online First: 2021/01/08]

8. Nay Yi Yi Linn, Tripathy JP, Maung TM, et al. How are the village health volunteers deliver malaria testing and treatment services and what are the challenges they are facing? A mixed methods study in Myanmar. *Trop Med Health* 2018;46:28-28. doi: 10.1186/s41182-018-0110-0

9. McLean ARD, Wai HP, Thu AM, et al. Malaria elimination in remote communities requires integration of malaria control activities into general health care: an observational study and interrupted time series analysis in Myanmar. *BMC Medicine* 2018;16(1):183. doi: 10.1186/s12916-018-1172-x

10. Myanmar National Malaria Control Programme. Natiaonl Plan for malaria elimination in Myanmar (2016 - 2030). In: Health DoP, ed. 1 ed. Nay Pyi Taw, Myanmar: National Malaria Control Programme, Department of Public Health, Ministry of Health and Sports, The Republic of the Union of Myanmar, 2017:74.

11. Myanmar National Malaria Control Programme. National strategic plan for malaria elimination 2021-2025 (Draft). In: Health DoP, ed. 2021 - 2025 ed. Nay Pyi Taw, Myanmar: Ministry of Health and Sports, Republic of the Union of Myanmar, 2019:63.

12. Abegunde D, Orobaton N, Bassi A, et al. The Impact of Integrated Community Case Management of Childhood Diseases Interventions to Prevent Malaria Fever in Children Less than Five Years Old in Bauchi State of Nigeria. *PloS one* 2016;11(2):e0148586. doi: 10.1371/journal.pone.0148586 [published Online First: 2016/02/06]

13. Brenner JL, Kabakyenga J, Kyomuhangi T, et al. Can volunteer community health workers decrease child morbidity and mortality in southwestern Uganda? An impact evaluation. *PloS one* 2011;6(12):e27997. doi: 10.1371/journal.pone.0027997 [published Online First: 2011/12/24]

14. Mubiru D, Byabasheija R, Bwanika JB, et al. Evaluation of Integrated Community Case Management in Eight Districts of Central Uganda. *PloS one* 2015;10(8):e0134767. doi: 10.1371/journal.pone.0134767 [published Online First: 2015/08/13]

15. Mukanga D, Tibenderana JK, Peterson S, et al. Access, acceptability and utilization of community health workers using diagnostics for case management of fever in Ugandan children: a cross-sectional study. *Malar J* 2012;11:121. doi: 10.1186/1475-2875-11-121 [published Online First: 2012/04/24]

16. Miller NP, Amouzou A, Tafesse M, et al. Integrated community case management of childhood illness in Ethiopia: implementation strength and quality of care. *The American journal of tropical medicine and hygiene* 2014;91(2):424-34. doi: 10.4269/ajtmh.13-0751 [published Online First: 2014/05/07]

17. Moe Myint Oo. Integrated Community Case Management Dissemination and Consultation Meeting. Meeting Hall, Disease Control Office, MoHS, Nay Pyi Taw: Malaria Consortium, 2017:51.

18. Myanmar Department of Public Health. Integrated Community Malaria Volunteer Manual. In: Health DoP, ed. First ed. Nay Pyi Taw: Myanmar Ministry of Health and Sports, 2017:93.

19. Myanmar Ministry of Health and Sports. Comprehensive literature review on village based health workers in Myanmar: extending services to communities Nay Pyi Taw, Myanmar Myanmar Ministry of Health and Sports, 2017:52.

20. Win Han Oo, Gold L, Moore K, et al. The impact of community-delivered models of malaria control and elimination: a systematic review. *Malar J* 2019;18(1):269. doi: 10.1186/s12936-019-2900-1 [published Online First: 2019/08/08]

21. Win Han O, Hoban E, Gold L, et al. Optimizing Myanmar’s community-delivered malaria volunteer model: a qualitative study of stakeholders’ perspectives. *Malaria Journal* 2021;20(1):79. doi: 10.1186/s12936-021-03612-6

22. Hemming K, Haines TP, Chilton PJ, et al. The stepped wedge cluster randomised trial: rationale, design, analysis, and reporting. *BMJ : British Medical Journal* 2015;350:h391. doi: 10.1136/bmj.h391

23. Agius PA, Cutts JC, Han Oo W, et al. Evaluation of the effectiveness of topical repellent distributed by village health volunteer networks against Plasmodium spp. infection in Myanmar: A stepped-wedge cluster randomised trial. *PLoS Med* 2020;17(8):e1003177. doi: 10.1371/journal.pmed.1003177 [published Online First: 2020/08/21]

24. Hemming K, Lilford R, Girling AJ. Stepped-wedge cluster randomised controlled trials: a generic framework including parallel and multiple-level designs. *Stat Med* 2015;34(2):181-96. doi: 10.1002/sim.6325 [published Online First: 2014/10/28]
