# Supplementary material for: Effectiveness of an expanded role for community health workers on malaria blood examination rates in malaria elimination settings in Myanmar: an open stepped-wedge, cluster-randomised controlled trial
Source: Lancet Reg Health Southeast Asia. 2024 Oct 17;31:100499. doi: 10.1016/j.lansea.2024.100499 (PMC11531616; doi:10.1016/j.lansea.2024.100499)
Supplement: Supplementary Material S2 [file mmc2.docx]

**
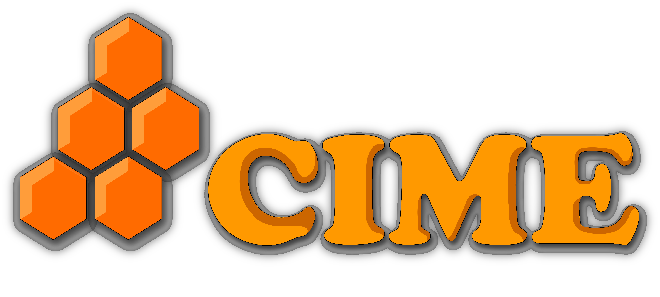
**

**Community-Delivered Integrated Malaria Elimination Model**

**Communit Health Worker Training**

**Curriculum**

**National Malaria Control Programme, Ministry of Health and Sports, Myanmar**

**&**

**Burnet Institute**

**September 2020**

**
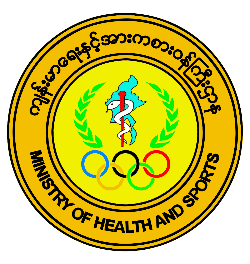
**

**
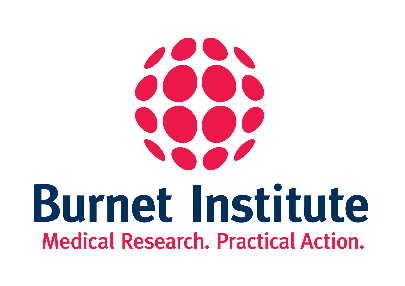
**

**Community-Delivered Integrated Malaria Elimination Model**

**Supplementary Material 2: Community Health Worker Training**

**Curriculum**

**National Malaria Control Programme, Ministry of Health and Sports, Myanmar**

**&**

**Burnet Institute**

**September 2020**

# About this Document

This is the curriculum developed for Community-Delivered Integrated Malaria Elimination (CIME) Model, Volunteer Training which is done as a part of the research study *“Evaluation of the implementation effectiveness and cost-effectiveness of the Community-delivered Integrated Malaria Elimination (CIME) model: Myanmar”*. This is the overview of the training curriculum and this document is to be used in combination with *“Community-Integrated Malaria Elimination Model, Volunteer Training: Facilitator’s Manual and Volunteer’s Manual”*.

# Contents

[About this Document 3](#_Toc56583399)

[Contents 4](#_Toc56583400)

[1. Background 5](#_Toc56583401)

[1.1. Training philosophy 5](#_Toc56583402)

[1.2. Target audience 6](#_Toc56583403)

[2. Training Objectives 7](#_Toc56583404)

[2.1. General objective 7](#_Toc56583405)

[2.2. Specific objectives 7](#_Toc56583406)

[3. Program Outline 8](#_Toc56583407)

[3.1. Training setting 8](#_Toc56583408)

[3.2. Contents and time allocation 8](#_Toc56583409)

[3.3. Strategies and methods 11](#_Toc56583410)

[3.4. Training materials 11](#_Toc56583411)

[3.5. Assessment and evaluation 12](#_Toc56583412)

[4. Post-training plan 13](#_Toc56583413)

[5. Reporting 13](#_Toc56583414)

[6. Funding and support 13](#_Toc56583415)

# Background

In the context of lower malarial incidence, increasing the health services that the malaria volunteers can provide, increases their utilization, and enables more malaria testing. With such a purpose, the Myanmar National Malaria Control Programme (NMCP) has rolled out the Integrated Community Malaria Volunteer (ICMV) program throughout Myanmar in 2017-18. In 2019-2020, Burnet Institute has performed a preliminary qualitative study of key stakeholders and community members assessing their preferred community-delivered models for malaria elimination and primary health care in rural communities of Myanmar so that components of the model can be chosen in an evidence-based approach. From the evidence gathered in this study, the Burnet Institute has produced an optimal community-delivered package, the Community-delivered Integrated Malaria Elimination (CIME) model for Myanmar which integrates interventions for malaria, dengue, tuberculosis, childhood diarrhoea, and RDT-negative fever.

At this point, it needs to assess the effectiveness and cost-effectiveness of the CIME model in delivering services compared to the currently running ICMV model, and for this purpose, the study ***“Evaluation of the implementation effectiveness and cost-effectiveness of the Community-delivered Integrated Malaria Elimination (CIME) model: Myanmar”*** is done. This involves comparing the CIME and ICMV models in terms of their acceptability and utilization by community members, fidelity and feasibility of the CIME model, quality of the reported data, and cost-effectiveness analysis. In this study, we introduce the CIME model to selected villages that are currently running with the ICMV model, by giving CIME Volunteer Training and subsequent supports to the ICMVs of these villages. After the training, the volunteers continue their work in their villages as CIME Volunteers until the end of this study.

Therefore, for effective and efficient training of the CIME Volunteers, this training curriculum ***“Community-Delivered Integrated Malaria Elimination Model, Volunteer Training Curriculum”*** is developed.

## Training philosophy

The CIME Volunteer Training intends to give birth to proficient and proactive CIME Volunteers who have sufficient knowledge and skill in the management of malaria, dengue, tuberculosis, childhood diarrhea, and RDT-negative fever in the community setting, through intensive training with various participatory learning activities. Through the provision of integrated primary health services for these diseases, the CIME Volunteers will initiate malaria elimination activities in their respective communities.

## Target audience

This training curriculum is targeted for the ICMVs in the NMCP network currently working in Yangon Region who will be transformed into CIME Volunteers after completion of this training.

As ICMVs, they have received training for malaria, dengue, filariasis, tuberculosis, HIV/AIDS, and leprosy. And almost all of them are currently actively working for malaria, and have at-least-working knowledge and skill for malaria testing with RDT, treatment of malaria cases and/or referral, malaria case notification, and behavioural change communication for malaria. As currently functioning ICMVs, the training participants are expected to be not too young or not too old, and at least able to read and write.

# Training Objectives

## General objective

To give birth of proficient and proactive CIME Volunteers who provide malaria services, in integration with services for dengue, tuberculosis, childhood diarrhea and RDT-negative fever, ultimately leading to a performance for malaria elimination better than before.

## Specific objectives

At the end of the training, the CIME Volunteers should:

1. Clearly know the roles and responsibilities of a CIME Volunteer;
2. Have knowledge and skill on malaria, dengue, tuberculosis, childhood diarrhea, and RDT-negative fever sufficient enough to work as a CIME Volunteer;
3. Have sufficient knowledge and skill on behavioral change communication (BCC) for these diseases;
4. Have sufficient knowledge and skill on recording and reporting of these diseases; and
5. Have sufficient knowledge, attitude, and skill to initiate malaria elimination activities in their respective communities by providing integrated services for these diseases.

# Program Outline

## Training setting

Recruitment of training participants is according to the research study protocol. The randomly selected 74 training participants are randomized into 10 groups which will include ICMVs from three townships of Yangon region. And each group receives the training at different times, fortnightly after another. The ICMVs of the same group are invited to the respective township health departments for the CIME Volunteer Training.

The total training duration is 28 hours over four days (with seven hours on each day) (with a lunch break and tea-/coffee-breaks in between the sessions). All training sessions are to be conducted in a well-accommodated comfortable room of each township -health department of the respective townships. The rooms should be well supported with appropriate furniture and a well-functioning PA system.

Training sessions are to be arranged and delivered by the Training Team which is composed of proficient staff from Burnet Institute, Myanmar (BIMM), and - State/Regional NMCP, who are well-experienced with different thematic areas of CIME diseases and well-orientated for CIME Volunteer training. A preparatory workshop of training facilitators is to be conducted before the proper CIME Volunteer Trainings.

## Contents and time allocation

The choice of contents, emphasis of topics, and time distribution for each subject matter and for each topic depend on the following assumptions and considerations:

- All trainees, as ICMVs, have already received trainings on malaria, dengue and TB and they are currently practicing these diseases, especially malaria.
- With malaria, the trainees have already had enough knowledge and skill to operate as a CIME Volunteer except for some malaria surveillance matters like malaria case investigation. This training is supposed to upgrade the malaria volunteers to another higher level.
- Operational emphasis of a CIME Volunteer has a greater weightage on malaria activities.
- Operational responsibilities of a CIME Volunteer for malaria and dengue will be higher than those of an ICMV.
- CIME Volunteer training is part of a research study.

The CIME Volunteers will be trained for the following contents. (For a detailed description of the course content, structure and sequence, see ***“Community-Integrated Malaria Elimination Model, Volunteer Training: Facilitator’s Manual and Volunteer’s Manual”***).

- - 1. CIME Model and CIME Volunteer (Duration: 0:30 hours)
- Introduction to CIME Model and CIME Volunteer
- Roles of CIME Volunteers (roles and responsibilities, supported package, do’s and don’ts)
  - 1. Malaria (Duration: - 5:45 hours)
- Current situation of malaria
- Basic knowledge and skill for malaria elimination (nature of malaria, transmission and lifecycle, diagnosis, malaria case management, malaria prevention, malaria surveillance, prevention of malaria outbreak)
- Role of CIME Volunteers in malaria elimination
  - 1. Dengue (Duration: - 1:30 hours)
- Current situation of dengue
- Basic knowledge and skill for dengue (nature of dengue, transmission, diagnosis, assisted referral, prevention and control)
- Role of CIME Volunteers in prevention and control of dengue
  - 1. Tuberculosis (Duration: - 2:45 hours)
- Current situation of tuberculosis
- Basic knowledge and skill for tuberculosis (nature of tuberculosis, transmission, diagnosis, assisted referral, treatment, prevention and control)
- Role of CIME Volunteers in prevention and control of tuberculosis
  - 1. Childhood diarrhea (Duration: 1:45 hours)
- Current situation of childhood diarrhea
- Basic knowledge and skill for childhood diarrhea (nature of childhood diarrhea, transmission, diagnosis, assisted referral, prevention and control)
- Role of CIME Volunteers in prevention and control of childhood diarrhea
  - 1. RDT-negative fever (Duration: - 2:45 hours)
- Basic knowledge and skill for RDT-negative fever (differential diagnosis of fever, assisted referral of a fever case, fever management)
- Role of CIME Volunteers in handling a fever case
  - 1. Behavioral change communication (Duration: 2:00hours)
- Basic knowledge and skill for behavioral change communication (importance of BCC, basic concepts of communication, interpersonal communication, basic concepts of BCC, how to conduct a BCC)
- BCC activities of CIME Volunteers
- Community advocacy of CIME Volunteer activities
- Handling refusals of CIME Volunteer services
  - 1. Recording and reporting, and logistics management (Duration: 2:00 hours)
- Importance of recording and reporting
- Records and reports for CIME Volunteers (Carbonless Malaria Register, CIME Volunteer Record Book, ICMV Daily Register, Patient Referral Form, monitoring and supervision)
- Logistic management
  - 1. Community-based health volunteers in the time of **COVID-19** (Duration: 1:00 hours)
    2. Prevention of Sexual Exploitation and Abuse Policy (Duration : 0:10 hours)

A total of 1:45 hours is used for pre- and post-training assessments, and overall discussion and evaluation. And, 1:00 hours are for introductory activities and conclusion. Lunch breaks and tea/coffee breaks take the rest of the time (5:00 hours).

(In this research study, in addition to the *CIME Volunteer Training* which is the introducing action of the intervention phase, the volunteers also receive a 2-hour training on recording and reporting of the whole study period. It happens at the start of the six-month study period before CIME Volunteer Training.)

This is the overview of the training agenda over the course of 4 days.

| Day | Time | Subject |
| --- | --- | --- |
| Day 1 | Morning | Opening, Introduction, Pretest & Malaria |
|  | Evening | Malaria |
| Day 2 | Morning | Malaria & Dengue |
|  | Evening | Tuberculosis |
| Day 3 | Morning | Childhood diarrhoea |
|  | Evening | RDT-negative fever |
| Day 4 | Morning | BCC and Recordings & Reporting |
|  | Evening | Notable things for CIME Volunteers, PSEA, Post test & Evaluation, & Closing |

## Strategies and methods

This training will use a combination of different strategies and methods that address all three domains of learning_ cognitive, affective, and psychomotor domains. For the acquisition of required knowledge and development of mental skills related to the CIME diseases, lectures, interactive discussion, brainstorming, problem analysis, critical thinking, and problem-solving are to be used. For the positive build-up of their feelings, emotions, and attitudes as a CIME Volunteer life, a combination of interactive discussion, roleplaying, experience sharing, storytelling, problem analysis and problem-solving, and setting their own standard of practice are to be used. For developing and strengthening their skills in case management, demonstration, hands-on exercise, roleplaying, and simulation exercise are to be used.

## Training materials

For effective delivery of the training, the following materials are to be used_ a well-functioning electronic audio-visual system including a laptop, an LCD projector and screen, and a PA system; *“Community-Integrated Malaria Elimination Model, Volunteer Training: Facilitator’s Manual* and *Volunteer’s Manual”* as handbooks for the trainers and trainees respectively; PowerPoint presentation slides; other required teaching aids such as medicines and equipment, and samples of recording and reporting forms; preset problems, quizzes and case scenarios; paper documents such as pre- and post-test questions, and evaluation forms; and other supporting materials like flip charts, markers, and other stationaries.

For a detailed list of training materials, see ***Appendix 2*** of ***“Community-Integrated Malaria Elimination Model, Volunteer Training: Facilitator’s Manual”***.

## Assessment and evaluation

Evaluation of the CIME Volunteers’ achievement is to be done by the Training Team. It will be done by Pre- and post-training assessments; performance of the volunteers in their practical and participatory learning activities; and interactive discussion in each training session.

The CIMV Volunteer with less than 50% achievement in the post-training assessment is taken as having unsatisfactory achievement. For CIME Volunteers who have not shown satisfactory achievement will be supported with further on-site training by their field supervisors during their field practice.

The overall achievement of the whole training event is to be evaluated by an overall discussion and a post-training evaluation form that addresses topics covered by the training, competency of the trainers, training methods, training logistic support (transportation and accommodation support, training venue, and refreshment) and the trainees’ overall satisfaction.

The results of all formative and summative assessments and evaluation will be used for improvement of the current training session as well as the next sessions.

# Post-training plan

After the training, the ICMVs will continue to work in their villages as CIME Volunteers. They will be continuously supported with material and technical support which will be mainly provided through the state/regional and township NMCP during their monitoring and supervision visits.

# Reporting

At the end of each individual training, a proper Training Report (including the training’s achievements and challenges) is to be prepared and disseminated to the respective members of BIMM and National and State/Regional NMCP (especially to the members of the Training Team). The findings and lessons learned from the accomplished trainings are to be applied to the next trainings. Significant findings of these reports are to be taken into account for the evaluation and modification of this curriculum in the future.

# Funding and support

This training is funded by the Regional Artemisinin-resistance Initiative 2 Elimination (RAI2E) Programme. Technical support and technical resource persons are supported by Myanmar National Malaria Control Programme (NMCP) and Burnet Institute.
