# Supplementary material for: Effectiveness of an expanded role for community health workers on malaria blood examination rates in malaria elimination settings in Myanmar: an open stepped-wedge, cluster-randomised controlled trial
Source: Lancet Reg Health Southeast Asia. 2024 Oct 17;31:100499. doi: 10.1016/j.lansea.2024.100499 (PMC11531616; doi:10.1016/j.lansea.2024.100499)
Supplement: Supplementary Material S3 [file mmc3.docx]

# Supplementary Material 3: Supervision and field observation checklists

## Supervision checklist

| **Supervision Checklist for Community Based Integrated Malaria Elimination (CIME) Volunteer Activities** |
| --- |

This is the supervision checklist for CIME volunteer’s supervisors. The supervision is expected to be conducted in about 2 hours.

| **1. Background Information** | | | | | |
| --- | --- | --- | --- | --- | --- |
| 1.1. | State/ Region: |  | 1.2. | Township: |  |
| 1.3. | Name of volunteer: |  | 1.4. | Village name: |  |
| 1.5. | Name of RHC: |  | 1.6. | Name of sub-center |  |
| 1.7. | Starting time: |  | 1.8. | Ending time: |  |
| 1.9. | Date of supervision (DD/MM/YYYY): |  | | | |
| **Setting** | | | | | |
| 1.10. | CIME signboard in visible place | Yes 🞎 No 🞎  🞎 Not seen |  | | |
| 1.11. | Where does CIME volunteer usually see the patient? | 🞎 Adequate ventilation  🞎 Adequate lighting  🞎 Adequate privacy  🞎 Other ........................................................................ | | | |

| **2. Records, Reports and References** | | | | |
| --- | --- | --- | --- | --- |
|  | | Items | | Reasons for “Absent” |
| 2.1. | Presence of records, reports and references | 1. 🞎 CIME volunteer manual 2. 🞎 Malaria carbonless register 3. 🞎 ICMV daily register 4. 🞎 CIME record book 5. 🞎 Referral form 6. 🞎 Malaria case investigation and classification form 7. 🞎 ICMV quarterly report 8. 🞎 Other …………………… | |  |
| Record reviewing for previous 2 weeks | | | | |
| Malaria Carbonless Register | | Number | Remarks | |
| 2.2. | Number of patients RDT tested |  |  | |
| 2.3. | Number of P.f positive patient |  |  | |
| 2.4. | Number of P.v positive patient |  |  | |
| 2.5. | Number of Mixed patients |  |  | |
| 2.6. | Number of malaria patient referral |  |  | |
| 2.7. | Reasons for referral | 🞎 Danger signs 🞎 Pregnant mother  🞎 Infant 🞎 Other ........................ | | |
| ICMV Daily Register | | Number | Remarks | |
| 2.8. | Number of patients attended |  |  | |
| 2.9. | Number of RDT (-ve) fever |  |  | |
| 2.10. | Number of Childhood Diarrhoea |  |  | |
| 2.11. | Number of TB suspected patient |  |  | |
| 2.12. | Number of Dengue suspected patient |  |  | |
| 2.13. | Number of referrals made |  | If all suspected cases are not referred, please ask the reasons and describe the solutions made.  ………………………………………………………  ………………………………………………………  ……………………………………………………… | |
| CIME record book | | Number | Remarks | |
| 2.14. | Number HE session |  |  | |
| 2.15. | Number of participants attended in HE session |  |  | |
| 2.16. | Number of malaria case notifications within 24 hours |  | If all malaria positive cases are not notified within 24 hours, please ask the reasons and solution made.  …………………………………………………  …………………………………………………  ………………………………………………… | |
| 2.17. | Number of initial case investigations and classification by CIME |  | If all malaria positive cases are not investigated, please ask the reasons and describe the solution made.  ………………………………………………....  …………………………………………………  ………………………………………………… | |
| 2.18. | Number of malaria patient provided DOT |  |  | |
| 2.19. | All records are used correctly. | Yes 🞎 No 🞎 | Which records are not used correctly? How the records are not used correctly? | |
| 2.20. | Submit reports regularly | Yes 🞎 No 🞎 |  | |
| 2.21. | Records, reports and references are kept in safe place  (dedicated box or bag, place where the volunteer can only have access) | Yes 🞎 No 🞎 |  | |
| 2.22. | Any difficulties with recording and reporting | Yes 🞎 No 🞎 | Please mention. | |
| 2.23. | How do you solve as a supervisor? |  | | |

| **3. Logistic Management** | | | | | |
| --- | --- | --- | --- | --- | --- |
| Medicines and commodities | | Remaining balance | Expired date | Is there stock out during last 2 weeks? | If yes, how many days? |
| 3.1. | RDT |  |  | Yes 🞎 No 🞎 | days |
| 3.2. | RDT Ziplock bag |  |  | Yes 🞎 No 🞎 | days |
| 3.3. | ACT |  |  | Yes 🞎 No 🞎 | days |
| 3.4. | CQ |  |  | Yes 🞎 No 🞎 | days |
| 3.5. | PQ |  |  | Yes 🞎 No 🞎 | days |
| 3.6. | Paracetamol |  |  | Yes 🞎 No 🞎 | days |
| 3.7. | Multivitamin |  |  | Yes 🞎 No 🞎 | days |
| 3.8. | Zinc |  |  | Yes 🞎 No 🞎 | days |
| 3.9. | ORS |  |  | Yes 🞎 No 🞎 | days |
| 3.10. | Enough balance of RDT and antimalarial | Yes 🞎 No 🞎 | If the balances are not enough for next 2 weeks, please refill. | | |
| Recording | | Tick | How it is recorded and reasons for “No”. | | |
| 3.11. | Presence of Stock book | Yes 🞎 No 🞎 |  | | |
| 3.12. | Correctly recorded | Yes 🞎 No 🞎 |  | | |
| 3.13. | Recorded up to date | Yes 🞎 No 🞎 |  | | |
| 3.14. | Consistent with usage and patient | Yes 🞎 No 🞎 |  | | |
| 3.15. | Consistent with ground balance | Yes 🞎 No 🞎 |  | | |
| Proper storage | | Tick | If yes, corrections made by supervisor. | | |
| 3.16. | Store properly in safe place (Box, bag) | Yes 🞎 No 🞎 |  | | |
| 3.17. | Enough space to store the stocks (Shelf, box) | Yes 🞎 No 🞎 |  | | |
| 3.18. | Far from Heat, Sunlight, Rain/Humidity | Yes 🞎 No 🞎 |  | | |
| 3.19. | RDT and medicines are damaged and/or changed in color. | Yes 🞎 No 🞎 |  | | |
| 3.20. | Lancet and blood contaminated materials are disposed into the safety box properly | Yes 🞎 No 🞎 |  | | |
| Supported package | | | | | |
| Items | | Tick | Current situation and reasons | | |
| 3.21. | CIME Backpack/ Plastic box | Yes 🞎 No 🞎 |  | | |
| 3.22. | Thermometer | Yes 🞎 No 🞎 |  | | |
| 3.23. | Weighing machine | Yes 🞎 No 🞎 |  | | |
| 3.24. | Torch light | Yes 🞎 No 🞎 |  | | |
| 3.25. | Malaria treatment chart | Yes 🞎 No 🞎 |  | | |
| 3.26. | Flipchart and poster, pamphlet | Yes 🞎 No 🞎 |  | | |
| 3.27. | Pen to record on RDT | Yes 🞎 No 🞎 |  | | |
| 3.28. | Ziplock bag for RDT storage | Yes 🞎 No 🞎 |  | | |
| 3.29. | Code sticker for RDT | Yes 🞎 No 🞎 |  | | |

| **CIME volunteer’s knowledge assessment and observation** | | | | |
| --- | --- | --- | --- | --- |
| **4. Malaria** | | | | |
| 4.1. | Knowledge assessment | Volunteer’s response | | |
| 4.1.1. | Please mention signs and symptoms. |  | | |
| 4.1.2. | Please mention mode of transmission. |  | | |
| 4.1.3. | How can malaria be prevented? |  | | |
| 4.1.4. | Please mention the signs and symptoms of severe malaria. |  | | |
| 4.1.4. | What are the testing criteria for RDT testing |  | | |
| 4.2. | Observation of RDT testing | Correct | Incorrect | Not applicable |
| 4.2.2. | RDT preparation |  |  |  |
| 4.2.3. | Patient preparation |  |  |  |
| 4.2.4. | Blood collection & dispensing |  |  |  |
| 4.2.5. | Timing and reading results |  |  |  |
| 4.2.6. | Recording results |  |  |  |
| 4.2.7. | Disposal of infectious material |  |  |  |
| 4.2.8. | Delivering result |  |  |  |
| 4.3. | Observation of Case Management | Yes | No | Not applicable |
| 4.3.1. | Assess the signs and symptoms of malaria |  |  |  |
| 4.3.2. | Assess the all signs of severe malaria |  |  |  |
| 4.3.3. | RDT testing |  |  |  |
| 4.3.4. | Giving the correct treatment |  |  |  |
| 4.3.5. | Counsel (correct messages on full dose, full course) |  |  |  |
| 4.3.6. | Refer to health facility if there is signs and symptoms of severe malaria, infant and pregnant mother |  |  |  |
| 4.3.7 | Notify malaria positive cases within 24 hours |  |  |  |
| 4.3.8 | Conduct initial malaria case investigation and classification by CIME volunteer |  |  |  |
| 4.3.9. | Conduct DOT for malaria positive patient |  |  |  |
| 4.4. | Questions for Malaria treatment technical competencies | Correct | Incorrect | Not applicable |
| 4.4.1 | A 36 years old man comes to you because of fever. The RDT test result shows PF and PV mixed infection. What will be the treatment for him? |  |  |  |
| 4.4.2 | A four years old boy presented to you with fever. The RDT test shows positive PV infection. What will be the treatment for him? |  |  |  |
| 4.4.3 | A 12-year-old girl presented to you for fever and the RDT test results show positive PF infection. What will be the treatment for her? |  |  |  |
| 4.4.4 | A pregnant mother comes to you for fever and the RDT results show positive PF infection. What will you do? |  |  |  |

| **5. Dengue** | | | | |
| --- | --- | --- | --- | --- |
| 5.1. | Knowledge assessment | Volunteer’s response | | |
| 5.1.1. | Please mention signs and symptoms. |  | | |
| 5.1.2. | Please mention mode of transmission. |  | | |
| 5.1.3. | How can dengue be prevented? |  | | |
| 5.1.4. | Please mention the Danger signs of Dengue. |  | | |
| 5.2. | Observation of Case Management | Yes | No | Not applicable |
| 5.2.1. | Assess the signs and symptoms of dengue |  |  |  |
| 5.2.2. | Assess the danger signs |  |  |  |
| 5.2.3. | Measure body temperature |  |  |  |
| 5.2.4. | Give paracetamol according to age |  |  |  |
| 5.2.5. | Give ORS according to age |  |  |  |
| 5.2.6. | Counsel (correct messages on danger signs, feeding, personal protective measures, larva control) |  |  |  |
| 5.2.7. | Refer to health facility |  |  |  |

| **6. Tuberculosis** | | | | |
| --- | --- | --- | --- | --- |
| 6.1. | Knowledge assessment | Volunteer’s response | | |
| 6.1.1. | Please mention signs and symptoms. |  | | |
| 6.1.2. | Please mention mode of transmission. |  | | |
| 6.1.3. | How can TB be prevented? |  | | |
| 6.1.4. | What are the treatment options for TB? |  | | |
| 6.2. | Observation of Case Management | Yes | No | Not applicable |
| 6.2.1. | Assess the signs and symptoms of TB |  |  |  |
| 6.2.2. | Assess history of suspected TB patient in family members |  |  |  |
| 6.2.3. | Measure body temperature |  |  |  |
| 6.2.4. | Give paracetamol according to age |  |  |  |
| 6.2.5. | Counsel (correct messages on signs and symptoms, transmission, treatment and preventive measures) |  |  |  |
| 6.2.6. | Refer to health facility |  |  |  |

| **7. Childhood diarrhea** | | | | |
| --- | --- | --- | --- | --- |
| 7.1. | Knowledge assessment | Volunteer’s response | | |
| 7.1.1. | Please mention signs and symptoms. |  | | |
| 7.1.2. | Please mention mode of transmission. |  | | |
| 7.1.3. | How can diarrhoea be prevented? |  | | |
| 7.1.4. | How to provide ORS and Zinc according to age. |  | | |
| 7.2. | Observation of Case Management | Yes | No | Not applicable |
| 7.2.1. | Assess the signs and symptoms of diarrhea. |  |  |  |
| 7.2.2. | Assess signs of dehydration |  |  |  |
| 7.2.3. | Give Zinc according to age |  |  |  |
| 7.2.4. | Give ORS according to age |  |  |  |
| 7.2.5. | Counsel (correct messages on signs and symptoms, transmission and preventive measures) |  |  |  |
| 7.2.6. | Refer to health facility |  |  |  |

| **8. RDT (-) ve Fever** | | | | |
| --- | --- | --- | --- | --- |
| 8.1. | Knowledge assessment | Volunteer’s response | | |
| 8.1.1. | Please mention causes of fever |  | | |
| 8.1.2. | Please mention measures to relieve fever |  | | |
| 8.2. | Observation of Case Management | Yes | No | Not applicable |
| 8.2.1. | Give Paracetamol according to age |  |  |  |
| 8.2.2. | Counsel (correct messages on causes of fever, measures to relieve fever) |  |  |  |
| 8.2.3. | Refer to health facility |  |  |  |

| **8. Findings and solution made by supervisors** | |
| --- | --- |
| **Findings** | **Solution made** |
| **……………………………………………**  **……………………………………………**  **……………………………………………**  **……………………………………………**  **……………………………………………**  **……………………………………………** | **……………………………………………**  **……………………………………………**  **……………………………………………**  **……………………………………………**  **……………………………………………**  **……………………………………………** |

Remarks: Please bring RDT, antimalarials, ORS, Zinc, Paracetamol, Pamphlet and Poster when you make supervision visit to volunteer.

Supervisor signature: ………………………

Supervisor name: ………………………….

Supervisor position: ………………………

Department: ………………………………

Date: ………………………………………

Volunteer signature: ………………………

Volunteer name: ………………………….

Village: ………………………………

Date: ………………………………………

## Field observation guide

This is the observation guide for CIME volunteer’s works. The observation will be conducted in volunteer’s place by the observer for the whole day.

| 1. **Background Information** | | | | | |
| --- | --- | --- | --- | --- | --- |
| 1.1. | State/ Region: |  | 1.2. | Township: |  |
| 1.3. | Name of RHC: |  | 1.4. | Name of sub-center |  |
| 1.5. | Village name: |  | 1.6. | Date of supervision (DD/MM/YYYY): |  |
| 1.7. | Starting time: |  | 1.8. | Ending time: |  |
| 1.9. | Name of observer: |  | | | |

| 1. **Village/Worksite profile** | | | | | | |
| --- | --- | --- | --- | --- | --- | --- |
| 2.1. | Socioeconomic status |  | | | | |
| 2.2. | Geography and topography | 🞎 Plain  🞎 Mountain Foot  🞎 Hill/ Slope  🞎 Near paddy filed | | 🞎 Near Dam/ stream/ river  🞎 Forest fringes  🞎 Other ....................................... | | |
| 2.3. | Available transportation mode | 🞎 Earthen road 🞎 Stone road 🞎 Paved road | | | | |
| 2.4. | Mobile network coverage | 🞎 Yes 🞎 No | If “Yes” Is the connection good? | | 🞎 Good 🞎 Poor | |
| 2.5. | Electricity source | 🞎 National grid 🞎 Solar 🞎 Generator 🞎 Personal Hydropower  🞎 Other ........................................................................... | | | | |
| 2.6. | Is there any government health facility? | 🞎 Yes 🞎 No | | | | |
| 2.7. | Distance from health facility | By vehicle | ...................... min/ hour | | | |
|  |  | By foot | ...................... min/ hour | | | |
| 2.8. | Is there any private clinic? | 🞎 Yes 🞎 No | If “Yes” how many? | | | ...................... |
| 2.9. | Is there any pharmacy? | 🞎 Yes 🞎 No | If “Yes” how many? | | | ...................... |

| 1. **Setting** | | | |
| --- | --- | --- | --- |
| 3.1. | CIME signboard in visible place | Yes 🞎 No 🞎  🞎 Not seen |  |
| 3.2. | Where does CIME volunteer usually see the patient? | 🞎 Adequate ventilation  🞎 Adequate lighting  🞎 Adequate privacy  🞎 Other ........................................................................ | |

| 1. **Records, Reports and References** | | | |
| --- | --- | --- | --- |
|  | | Items | Reasons for “Absent” |
| 4.1. | Presence of records, reports and references | 🞎 CIME volunteer manual  🞎 Malaria carbonless register  🞎 ICMV daily register  🞎 CIME record book  🞎 Referral form  🞎 Malaria case investigation and classification form  🞎 ICMV quarterly report  🞎 Other ……………………… |  |

| Record Reviewing (Please review the previous 6 months records) | | | | | | | | |
| --- | --- | --- | --- | --- | --- | --- | --- | --- |
| Malaria Carbonless Register | | Month 1 | Month 2 | Month 3 | Month 4 | Month 5 | Month 6 | Remarks |
| 4.2. | Number of patients RDT tested |  |  |  |  |  |  |  |
| 4.3. | Number of P.f positive patient |  |  |  |  |  |  |  |
| 4.4. | Number of P.f patient with correct treatment |  |  |  |  |  |  |  |
| 4.5. | Number of P.v positive patient |  |  |  |  |  |  |  |
| 4.6. | Number of P.v patient with correct treatment |  |  |  |  |  |  |  |
| 4.7. | Number of Mixed positive patient |  |  |  |  |  |  |  |
| 4.8. | Number of Mixed patient with correct treatment |  |  |  |  |  |  |  |
| 4.9. | Number of malaria patient referral |  |  |  |  |  |  |  |
| 4.10. | Reasons for referral | 🞎 Danger signs 🞎 Pregnancy 🞎 Under 1 year 🞎 Other ........................ | | | | | | |
| ICMV Daily Register | | Month 1 | Month 2 | Month 3 | Month 4 | Month 5 | Month 6 | Remarks |
| 4.11. | Number of patient attended |  |  |  |  |  |  |  |
| 4.12. | Number of RDT (-) ve fever |  |  |  |  |  |  |  |
| 4.13. | Number of Childhood Diarrhoea |  |  |  |  |  |  |  |
| 4.14. | Number of TB suspected patient |  |  |  |  |  |  |  |
| 4.15. | Number of Dengue suspected patient |  |  |  |  |  |  |  |
| 4.16. | Number of Filariasis suspected patient |  |  |  |  |  |  |  |
| 4.17. | Number of Leprosy suspected patient |  |  |  |  |  |  |  |
| 4.18. | Number of HIV/AIDS suspected patient |  |  |  |  |  |  |  |
| 4.19. | Number of Sexually Transmitted diseases suspected |  |  |  |  |  |  |  |
| 4.20. | Number of referrals made |  |  |  |  |  |  |  |
| CIME record Book | | Month 1 | Month 2 | Month 3 | Month 4 | Month 5 | Month 6 | Remarks |
| 4.21. | Number of HE session |  |  |  |  |  |  |  |
| 4.22. | Number of participants in HE session |  |  |  |  |  |  |  |
| 4.23. | Number of notifications within 24 hours |  |  |  |  |  |  |  |
| 4.24. | Number of initial case investigation and classification by CIME |  |  |  |  |  |  |  |
| 4.25. | Number of malaria patient provided DOT |  |  |  |  |  |  |  |

| Reviewing the recording, collection, storage and difficulties | | | |
| --- | --- | --- | --- |
| 4.26. | All records and reports are using correctly.  🞎 Malaria carbonless register | Yes 🞎 No 🞎 | If the records and reports are not used correctly, how?  …................................................……………………………………… |
|  | 🞎 ICMV daily register | Yes 🞎 No 🞎 | …................................................……………………………………… |
|  | 🞎 CIME record book | Yes 🞎 No 🞎 | …................................................……………………………………… |
|  | 🞎 Referral form (using referral form in every referral) | Yes 🞎 No 🞎 | …................................................……………………………………… |
|  | 🞎 Malaria case investigation and classification form | Yes 🞎 No 🞎 | …................................................……………………………………… |
|  | 🞎 ICMV quarterly report | Yes 🞎 No 🞎 | …................................................……………………………………… |
|  | 🞎 Other …………………… | Yes 🞎 No 🞎 | …................................................……………………………………… |
| 4.27. | How the records and reports are collecting or submitting? | 🞎 CIME volunteer go and submit to township focal  🞎 Respective township focal go and collect from CIME volunteer  🞎 Submit via someone  🞎 Other .......................................................................................................... | |
| 4.28. | How often the records and reports are collecting or submitting? | 🞎 fortnightly  🞎 Monthly  🞎 Two monthlies  🞎 Quarterly  🞎 Other .............. | If there is no regular collecting or submitting, which records and reports?  …................................................………………………………………  …................................................………………………………………. |
| 4.29. | The records and reports are kept in safe place.  (dedicated box or bag, place where the volunteer can only have access) | Yes 🞎 No 🞎 | …................................................………………………………………  …................................................……………………………………… |
| 4.30. | Any difficulties with recording and reporting | Yes 🞎 No 🞎 | Please mention.  …................................................………………………………………  …................................................………………………………………  …................................................……………………………………… |

| 1. **Logistic Management (Please review stock in and out for previous six month** | | | | | | | | | | | |
| --- | --- | --- | --- | --- | --- | --- | --- | --- | --- | --- | --- |
| Medicines and commodities | | Remaining balance | | | In Total | Out  Total | | | Use  Total | Closing balance | Expired date |
| 5.1. | RDT |  | | |  |  | | |  |  |  |
| 5.2. | ACT 24 |  | | |  |  | | |  |  |  |
| 5.3. | ACT 18 |  | | |  |  | | |  |  |  |
| 5.4. | ACT 12 |  | | |  |  | | |  |  |  |
| 5.5. | ACT 6 |  | | |  |  | | |  |  |  |
| 5.6. | CQ |  | | |  |  | | |  |  |  |
| 5.7. | PQ |  | | |  |  | | |  |  |  |
| 5.8. | Paracetamol |  | | |  |  | | |  |  |  |
| 5.9. | Multivitamin |  | | |  |  | | |  |  |  |
| 5.10. | Zinc |  | | |  |  | | |  |  |  |
| 5.11. | ORS |  | | |  |  | | |  |  |  |
| 5.12. | Is there any stock out? | | Yes 🞎 No 🞎 | | | | If “Yes”, Which drugs and how long? | | | | |
| 5.13. | There will be RDT stock out in coming one month.  (Current balance < AMC of last 3 months) | | Yes 🞎 No 🞎 | | | |  | | | | |
| 5.14. | There will be RDT overstock in coming months.  (Month of stock> Remaining month’s shelf life) | | Yes 🞎 No 🞎 | | | | Month of stock = Average stock ÷ AMC of last 3 months  Remaining month’s shelf life = expired date – date of observation) | | | | |
| 5.15 | Expired stocks are stored in dedicated box/place. | | Yes 🞎 No 🞎 | | | |  | | | | |
| 5.16. | Label on the RDT and medicines are able to seen. | | Yes 🞎 No 🞎 | | | |  | | | | |
| 5.17. | Volunteer is able to check the expiry date. | | Yes 🞎 No 🞎 | | | |  | | | | |
| 5.18. | How does the volunteer manage when the RDT and medicines are expired.  (Please ask the volunteer) | |  | | | | | | | | |
| Stock recording | | | |  | | | | How it is recorded and reasons for “No”. | | | |
| 5.19. | Presence of Stock book | | Yes 🞎 No 🞎 | | | |  | | | | |
| 5.20. | Correctly recorded | | Yes 🞎 No 🞎 | | | |  | | | | |
| 5.21. | Recorded up to date | | Yes 🞎 No 🞎 | | | |  | | | | |
| 5.22. | Consistent with usage and patient for last month | | Yes 🞎 No 🞎 | | | |  | | | | |
| 5.23. | Consistent with ground balance during observation | | Yes 🞎 No 🞎 | | | |  | | | | |
| Proper storage | | | |  | | | | If yes, corrections made by supervisor. | | | |
| 5.24. | Store properly in safe place (Box, bag) | | Yes 🞎 No 🞎 | | | |  | | | | |
| 5.25. | Enough space to store the stock (Shelf, Box) | | Yes 🞎 No 🞎 | | | |  | | | | |
| 5.26. | Far from Heat, Sunlight, Rain/Humidity | | Yes 🞎 No 🞎 | | | |  | | | | |
| 5.27. | RDT and medicines are damaged and/or changed in color. | | Yes 🞎 No 🞎 | | | |  | | | | |
| 5.28. | Lancet and blood contaminated materials are disposed into the Safety box properly | | Yes 🞎 No 🞎 | | | |  | | | | |
| 5.29. | How is the safety box disposed?  (Please ask the volunteer) | |  | | | | | | | | |
| Supported package | | | | | | | | | | | |
| Items | | | |  | | | | Current situation and reasons | | | |
| 5.30. | CIME Backpack/ Plastic box | | Yes 🞎 No 🞎 | | | |  | | | | |
| 5.31. | Thermometer | | Yes 🞎 No 🞎 | | | |  | | | | |
| 5.32. | Weighing machine | | Yes 🞎 No 🞎 | | | |  | | | | |
| 5.33. | Torch light | | Yes 🞎 No 🞎 | | | |  | | | | |
| 5.34. | Malaria treatment chart | | Yes 🞎 No 🞎 | | | |  | | | | |
| 5.35. | Flipchart, poster, pamphlet | | Yes 🞎 No 🞎 | | | |  | | | | |
| 5.36. | Pen to record on RDT | | Yes 🞎 No 🞎 | | | |  | | | | |
| 5.37. | Ziplock bag for RDT storage | | Yes 🞎 No 🞎 | | | |  | | | | |
| 5.38. | Code sticker for RDT | | Yes 🞎 No 🞎 | | | |  | | | | |

| 1. **Assessing the CIME volunteer’s service provision** | | | |
| --- | --- | --- | --- |
| 6. | Do you see any mentioned activities conducting by CIME volunteer during your observation | 🞎 Do not see any activity during observation  🞎 Case management, recording and referral activity  🞎 Malaria surveillance activity  🞎 Prevention and control, DOTS provider activity  🞎 Health education activity | |
| **Case Management** | | | |
| **6.1** | **Malaria** | | |
| History and examination | | | |
| 6.1.1. | Ask patient profile  (Name, Age, Sex, Pregnancy status, Address and Occupation) | Yes 🞎 No 🞎 |  |
| 6.1.2. | Assess malaria signs and symptoms | Yes 🞎 No 🞎 |  |
| 6.1.3. | Assess travelling history | Yes 🞎 No 🞎 |  |
| 6.1.4. | Asses Danger signs | Yes 🞎 No 🞎 |  |
| 6.1.5. | Measure body temperature correctly | Yes 🞎 No 🞎 |  |
|  | RDT testing | | |
| 6.1.6. | Check expiry and damage | Yes 🞎 No 🞎 |  |
| 6.1.7. | Record patient information on RDT | Yes 🞎 No 🞎 |  |
| 6.1.8. | Wear gloves | Yes 🞎 No 🞎 |  |
| 6.1.9. | Wipe with alcohol pad at finger tip | Yes 🞎 No 🞎 |  |
| 6.1.10. | Pierce with lancet and wipe first blood with clean cotton | Yes 🞎 No 🞎 |  |
| 6.1.11. | Take the blood with pipette (5 μl) | Yes 🞎 No 🞎 |  |
| 6.1.12. | Put the blood into the “S” correctly | Yes 🞎 No 🞎 |  |
| 6.1.13. | Put the 4 drops of buffer solution | Yes 🞎 No 🞎 |  |
| 6.1.14. | Record the time on RDT | Yes 🞎 No 🞎 |  |
| 6.1.15. | Read the result within 15 to 30 minutes | Yes 🞎 No 🞎 |  |
| 6.1.16. | Explain the result to patient | Yes 🞎 No 🞎 |  |
| 6.1.17. | RDT’s result | 🞎 P.f  🞎 P.v  🞎 Mixed  🞎 (-)ve |  |
| Treatment | | | |
| 6.1.18. | Correctly provide Treatment  P.f - ACT × 3 days and PQ × 1st day  P.v - CQ × 3 days and PQ × 14 days  Mixed - ACT × 3 days and PQ × 14 days  RDT (-)ve – Refer to BHS | Yes 🞎 No 🞎 |  |
| Health education | | | |
| 6.1.19. | Provide HE and counsel | Yes 🞎 No 🞎 | If Yes, please tick  🞎 Signs and symptoms  🞎 Transmission  🞎 Danger signs  🞎 How to administer the medicine  🞎 Importance of taking full course of medicine  🞎 DOTS  🞎 Preventive measures  🞎 Other, ……………………………… |
| **6.2** | **Dengue** | | |
| History and examination | | | |
| 6.2.1. | Ask patient profile  (Name, Age, Sex, Address) | Yes 🞎 No 🞎 |  |
| 6.2.2. | Assess signs and symptoms of Dengue | Yes 🞎 No 🞎 |  |
| 6.2.3. | Assess Danger signs | Yes 🞎 No 🞎 |  |
| 6.2.4. | Measure body temperature correctly | Yes 🞎 No 🞎 |  |
| Initial treatment | | | |
| 6.2.5. | Give paracetamol correctly according to age | Yes 🞎 No 🞎 |  |
| 6.2.6. | Give ORS correctly according to age | Yes 🞎 No 🞎 |  |
| Health education | | | |
| 6.2.7. | Provide HE and counsel | Yes 🞎 No 🞎 | If Yes, please tick  🞎 Sign and symptoms  🞎 Transmission  🞎 Danger signs  🞎 Personal protective measures  🞎 Larva control  🞎 Other, ……………………………… |
| **6.3** | **Tuberculosis** | | |
| History and examination | | | |
| 6.3.1. | Ask patient profile  (Name, Age, Sex, Address) | Yes 🞎 No 🞎 |  |
| 6.3.2. | Assess signs and symptoms of Tuberculosis | Yes 🞎 No 🞎 |  |
| 6.3.3. | Assess history of suspected TB patient in family members | Yes 🞎 No 🞎 |  |
| 6.3.4. | Measure body temperature correctly | Yes 🞎 No 🞎 |  |
| Initial treatment | | | |
| 6.3.5. | Give paracetamol correctly according to age | Yes 🞎 No 🞎 |  |
| Health education | | | |
| 6.3.6. | Provide HE and counsel | Yes 🞎 No 🞎 | If Yes, please tick  🞎 Sign and symptoms of TB  🞎 Transmission  🞎 Prevention of TB  🞎 Other, ……………………………… |
| **6.4** | **Childhood diarrhoea** | | |
| History and examination | | | |
| 6.4.1. | Ask patient profile  (Name, Age, Sex, Address) | Yes 🞎 No 🞎 |  |
| 6.4.2. | Assess signs and symptoms of diarrhea  (times, types) | Yes 🞎 No 🞎 |  |
| 6.4.3. | Assess signs of dehydration | Yes 🞎 No 🞎 |  |
| Initial treatment | | | |
| 6.4.4. | Provide Zinc correctly according to age | Yes 🞎 No 🞎 |  |
| 6.4.5. | Provide ORS correctly according to age | Yes 🞎 No 🞎 |  |
| Health education | | | |
| 6.4.6. | Provide HE and counsel | Yes 🞎 No 🞎 | If Yes, please tick  🞎 Signs and symptoms  🞎 Transmission  🞎 4 cleans  🞎 Benefits of ORS and Zinc in diarrhoea  🞎 How to prepare ORS solution  🞎 Preventive measures  🞎 Danger of antibiotics use  🞎 Other, ……………………………… |
| **6.5** | **RDT (-ve) Fever** | | |
| History and examination | | | |
| 6.5.1. | Ask patient profile  (Name, Age, Sex, Address) | Yes 🞎 No 🞎 |  |
| 6.5.2. | Ask history of fever | Yes 🞎 No 🞎 |  |
| 6.5.3. | Measure body temperature correctly | Yes 🞎 No 🞎 |  |
| Initial treatment | | | |
| 6.5.4. | Provide Paracetamol correctly according to age | Yes 🞎 No 🞎 |  |
| Health education | | | |
| 6.5.6. | Provide HE and counsel | Yes 🞎 No 🞎 | If Yes, please tick  🞎 Causes of fever  🞎 Measures to relieve fever  🞎 Other, ……………………………… |

| **6.6** | **Recording** | | |
| --- | --- | --- | --- |
| 6.5.1. | Record on the respective register correctly  🞎 Malaria carbonless register | Yes 🞎 No 🞎 |  |
|  | 🞎 ICMV daily register | Yes 🞎 No 🞎 |  |
|  | 🞎 CIME record book | Yes 🞎 No 🞎 |  |
|  | 🞎 Referral form | Yes 🞎 No 🞎 |  |
|  | 🞎 Malaria case investigation and classification form | Yes 🞎 No 🞎 |  |
|  | 🞎 ICMV quarterly report | Yes 🞎 No 🞎 |  |
|  | 🞎 Other ………………… | Yes 🞎 No 🞎 |  |
| **6.7** | **Referral** | | |
| 6.5.5. | Referral to BHS according to instruction  (Danger signs, Pregnancy and Under 1 children, TB, Dengue, Childhood diarrhea and RDT (-)ve fever) | Yes 🞎 No 🞎 |  |
| 6.5.6. | Information are correctly mentioned on referral form | Yes 🞎 No 🞎 |  |
| **6.8.** | **Malaria surveillance** | | |
| 6.8.1. | Notification within 24 hours | Yes 🞎 No 🞎 |  |
| 6.8.2. | Means of notification | 🞎 Voice call  🞎 SMS message  🞎 Viber or Messenger message  🞎 Other ……………………………………………. | |
| 6.8.3. | Any difficulties with notification | Please mention. | |
| 6.8.4. | Conduct initial malaria case investigation and classification | Yes 🞎 No 🞎 |  |
| 6.8.5. | Any difficulties with malaria case investigation and classification | 🞎 Section A: Basic information of patient  🞎 Section B: Case classification  🞎 Section C: Analysis of forward transmission  🞎 Section D: Reactive case detection  🞎 Section E: Case classification (Summary) | |
| 6.8.6. | If yes, please describe the difficulties |  | |
| 6.8.7. | Assist in Focus investigation and response | Yes 🞎 No 🞎 | How? |
| **6.9.** | **Prevention and control activities, DOTS providers** | | |
| 6.9.1. | Does the volunteer provide health education about preventive and control measures or distribute IECs? | Yes 🞎 No 🞎 | If yes, which measures? |
| 6.9.2. | What are the IECs materials available in the hands of volunteers? | 🞎 Poster  🞎 Pamphlet  🞎 Other, …………………………………………….. | |
| 6.9.3 | Other preventive activities carried out by the volunteer | 🞎 Assist in LLIN distribution  🞎 Assist in Indoor Residual Spraying  🞎 Larva control measures  🞎 Other, ………………………………………….. | |

| Larva control | | | |
| --- | --- | --- | --- |
| 6.9.4. | Carry out larva control measures | Yes 🞎 No 🞎 | Number of households conducted larva control measures |
| 6.9.5. | Activities of larva control measures |  | |
| Help in sputum collection and DOTS provider | | | |
| 6.9.6. | Help in sputum collection | Yes 🞎 No 🞎 | How? |
| 6.9.7. | CIME volunteer act as DOT supervisor | Yes 🞎 No 🞎 | If “Yes”, How many patients? |
| 6.9.8 | Provide anti TB medicine correctly | Yes 🞎 No 🞎 |  |

| **6.10.** | **Health Education** | | |
| --- | --- | --- | --- |
| Setting | | | |
| 6.10.1. | Place of HE |  | |
| 6.10.2. | Method of HE | 🞎 Group discussion  🞎 Lecture  🞎 Informal talk | |
| 6.10.3. | Material aids for HE | 🞎 Poster  🞎 Pamphlet  🞎 Flipchart  🞎 Other ……………………………………………… | |
| Audience | | | |
| 6.10.4. | Number of participants |  | |
| 6.10.5. | Type of participants  (Sex, Age Group, Migrant) |  | |
| Topic | | | Sub topic |
| 6.10.6. | 🞎 Malaria | | 🞎 Signs and symptoms  🞎 Transmission  🞎 Danger signs  🞎 Treatment options  🞎 Importance of taking full course of medicine  🞎 Preventive measures  🞎 Other, ………………………………… |
| 6.10.7. | 🞎 Dengue | | 🞎 Signs and symptoms  🞎 Transmission  🞎 Danger signs  🞎 Initial measures before arrival to health facility  🞎 Prevention and control measures  🞎 Larva control  🞎 Others, …………………………………. |
| 6.10.8. | 🞎 Tuberculosis | | 🞎 Signs and symptoms  🞎 Transmission  🞎 Diagnosis methods  🞎 Treatment options  🞎 Prevention and control measures  🞎 Others, …………………………………. |
| 6.10.9. | 🞎 Childhood diarrhoea | | 🞎 Signs and symptoms  🞎 Transmission  🞎 Danger signs  🞎 Initial measures before arrival to health facility  🞎 Benefits of ORS and Zinc  🞎 How to prepare ORS  🞎 Prevention and control measures  🞎 Danger of antibiotics misuse  🞎 Others, …………………………………. |
| 6.10.10. | Is the health education session provided by volunteer understandable? | | Please comment.  ………………………………………………  ……………………………………………… |
| 6.10.11. | Is the volunteer actively facilitate through the health education session? | | Please comment.  ………………………………………………  ……………………………………………… |
| End of session | | | |
