# Supplementary material for: Effectiveness of an expanded role for community health workers on malaria blood examination rates in malaria elimination settings in Myanmar: an open stepped-wedge, cluster-randomised controlled trial
Source: Lancet Reg Health Southeast Asia. 2024 Oct 17;31:100499. doi: 10.1016/j.lansea.2024.100499 (PMC11531616; doi:10.1016/j.lansea.2024.100499)
Supplement: Supplementary Material S4 [file mmc4.docx]

## Supplementary Material 4: Baseline data collection form

## Baseline data to be collected from the Yangon Vector Borne Diseases Control Unit/ Myanmar National Malaria Control Programme

| No. | Variable |
| --- | --- |
| 1 | General information of the village   - GPS coordinate of the village - MIMU name and P-code (if present) - Total number of households - Total number of population - Presence of migrant workers in the village (Yes/No) - Type and proportion of migrant workers in the village (if present) - Presence of worksite around the village (Yes/No) - Type and size of worksite around the village (if present) - Distance to the nearest health facility - Proximity to forested area (estimated miles) - Number of malaria implementing partners operating in the village - Number of volunteer currently active in the village |
| 2 | Malaria indicators   - Total number of RDT tested in 2019 (disaggregated by age group, sex) - Total number of malaria cases in 2019 (disaggregated by species, age group, sex) - Annual blood examination rate in 2019 - Annual parasite incidence in 2019 - Malaria test positive rate in 2019 |
| 3 | Information on ICMV/CIME volunteer   - Age - Sex - Volunteer start date - Date of MCBR training (if present) |
| 4 | Information on malaria preventive services   - Date of last LLIN mass distribution - Date of last IRS activity (if any) |

## Baseline information to be collected from the community health workers

| **No.** | **Question** | | | **Answer** | |
| --- | --- | --- | --- | --- | --- |
| 1.1 | Is there any mobile and migrant population in the village who came from other areas? | | | 1. Yes  2. No (SKIP to 1.2) | |
| 1.1.1 | What proportion of village population is mobile and migrant population from other areas? | | | ………………………% | |
| 1.2 | Is there any people from village who regularly travel to other regions/countries for work? | | | 1. Yes  2. No (SKIP to 1.3) | |
| 1.2.1 | What proportion of village population regularly travel to other regions/countries for work | | | ………………………% | |
| 1.3 | Is there any forest goers among village population? | | | 1. Yes  2. No (SKIP to 1.4) | |
| 1.3.1 | What proportion of village population is forest goer? | | | ………………………% | |
| 1.3.2 | What are the main purposes of forest going population? | | | 1. ……………………….  2. ……………………….  3. ……………………….  4. ……………………….  5. ………………………. | |
| 1.4 | What is the distance from the village to the nearest health facility? | | | …………… miles | |
| 1.5 | How much time does it take to go the nearest health facility using usual mode of transport? | | | ……hours …… minutes | |
| 1.6 | How do people from village go to the nearest health facility?  (Select all that apply) | | | 1. On foot  2. By motorcycle  3. By boat  4. Others (mention) | |
| 1.7 | Is there any ongoing armed conflict in the village and its surrounding of 1-mile radius? | | | 1. Yes  2. No (SKIP to 1.8) | |
| 1.7.1 | How many months has been passed since the start of current conflict? | | | …………… months | |
| 1.8 | Was there any armed conflict in the village and its surrounding of 1-mile radius in the past 3 years? | | | 1. Yes  2. No | |
| 1.9 | Is there any mobile phone signal in your village currently? | | | 1. Yes  2. No (SKIP to 1.10) | |
| 1.9.1 | Which operators (SIM cards) are working in your village?  (Select all that apply) | | | 1. MPT  2. Telenor  3. Ooredoo  4. MyTel  5. Others (mention) | |
| 1.9.2 | Please rate the quality of mobile phone signal in your village currently. | Good  (5 signal bars) | Fair  (3-4 signal bars) | Poor  (1-2 signal bars) | No service |
|  | MPT |  |  |  |  |
|  | Telenor |  |  |  |  |
|  | Ooredoo |  |  |  |  |
|  | MyTel |  |  |  |  |
|  | Others (mention) |  |  |  |  |
| 1.10 | Is there internet access in your village currently? | | | 1. Yes  2. No (END) | |
| 1.10.1 | Please rate the quality of internet access in your village currently. | | | 1. Good  2. Fair  3. Poor | |
