# Supplementary material for: Effectiveness of an expanded role for community health workers on malaria blood examination rates in malaria elimination settings in Myanmar: an open stepped-wedge, cluster-randomised controlled trial
Source: Lancet Reg Health Southeast Asia. 2024 Oct 17;31:100499. doi: 10.1016/j.lansea.2024.100499 (PMC11531616; doi:10.1016/j.lansea.2024.100499)
Supplement: Supplementary Material S5 [file mmc5.pdf]

Malaria Case Register for Volunteers

Stage/Region: **Township:tt** **Station/Rural Health Centre:** **Sub RHC:**

Organization: **Village:** **Name of Volunteer:** **Reporting month & year:**

Sr.	Date	Name	Age	Address	Sex		Pregnant	RDT results				Status		Antimalaria given						Referral to health facility	Malaria deaths	Seek treatment		History of travelling (within 2-4 weeks)	Occupation<br>1. Rubber plantation<br>2. Agriculture<br>3. Construction<br>4. Forestry<br>5. Mining<br>6. Others	Remark
					M	F		Positive			RDT negative	Not severe	Severe	ACT24	ACT18	ACT 12	ACT 6	Chloroquine	Primaquine			Within 24 hours	After 24 hours			
								P.f	*P.v*	Mix																

Month	Particular	Malaria RDT	ACT 24	ACT 18	ACT 12	ACT 6	Chloroquine	Primaquine
	Remaining from previous month							
	Received for this month							
	Total							
	This month usage							
	Closing balance							

Signature:

Name:

Village:

Organization:

Daily Register

Centre/Sub-centre ………………………. **Township …………………** **State/Region ………………………**

Sr.	Date	Name	Sex	Age	Address	Symptoms	RDT negative fever	Diarrhoea	Suspected						TB	DOTS	Educating filariasis patient with home care for secondary prevention	Leprosy complications	Number of condoms distributed	Referral	Health education
									TB	Dengue fever	Filariasis	Leprosy	HIV/AIDS	STD							

*** Daily register is confidential document and needs to be kept safely in appropriate place.

…Malaria data must be taken from carbonless register for malaria patients

…Tick only one disease in suspected column

Community-delivered integrated malaria elimination volunteer's record book

State/Region............................. Township... Station/rural health centre.. Sub-rural health centre..

Organisation - **NMCP** Village.. Name of volunteer... Reporting period from .... to .... Month.................................... Year..................................

(A) Community-delivered integrated malaria elimination volunteer's activity record

Date	Activities	Detailed description

Example activities

- Health education
- Bed net distribution
- Malaria case investigation
- Larva control activities
- Patient referral
- DOTS to TB cases
- Population and bed net data collection
- Assistance in vaccination

(B) Community-delivered integrated malaria elimination volunteer's special unique situation

(C) Malaria case notification and investigation records

No	Date	Name	Age	Sex	Address	Positive malaria species(P.f, P.v, Mix)	Case notification		Preliminary case investigation by volunteer (Yes/No)	DOTS (Yes/No)	Treatment completion (Yes/No/Cannot contact)	Re-testing with RDT		Remark
							Notify (Yes/No)	Notify within 24 hours (Yes/No)				Testing within 28 days (Yes/No)	RDT re-test result	
ɔ														

(D) Summary record of medicine and commodities usage

Description	Paracetamol (Tablet)	Paracetamol (Syrup)	Multi-vitamin	ORS	Zinc tablet
Previous remaining balance					
Received this month					
Total					
Use					
Current balance					

- RDT / medicines stock out (Yes/No) ....
- Total duration of stock out ....
- Stock out material ....

....

- Remark ....

Signature ....

Name of volunteer ....

Date ....

CIME Referral Form

Date ……………

Name ………………… Age ……. years Gender ………

Address ………

Referred health centre ……………………………………………………………………………………………

Reason for referral ……………………………………………………………………………………………...

Volunteer's

Signature …………….

Name ……………………….

Address ……………………….

(Receipt for volunteer)

CIME Referral Form

Date ……………

Name ………………… Age ……. years Gender ………

Address ………

Referred health centre ……………………………………………………………………………………………

Reason for referral ……………………………………………………………………………………………...

Volunteer's

Signature …………….

Name ……………………….

Address ……………………….

(Receipt for referred health centre)

CIME Referral Form

Date ……………

Name ………………… Age ……. years Gender ………

Address ………

Name of the volunteer ……………………………………………………………………………………………

Reason for referral ……………………………………………………………………………………………...

Diagnosis (To be filled by responsible health personnel) ……………………………………………………………
……..

Health personnel's

Signature …………….

Name ……………………….

Designation ……………………….

Facility ……………………….

(Receipt to send back to volunteer)
