# Supplementary material for: Effectiveness of an expanded role for community health workers on malaria blood examination rates in malaria elimination settings in Myanmar: an open stepped-wedge, cluster-randomised controlled trial
Source: Lancet Reg Health Southeast Asia. 2024 Oct 17;31:100499. doi: 10.1016/j.lansea.2024.100499 (PMC11531616; doi:10.1016/j.lansea.2024.100499)
Supplement: Supplementary Material S6 [file mmc6.docx]

# **Supplementary material 6 – Supplementary tables and figures**

## Supplementary Table 1: Number of community health workers (CHWs) who attended the Community-delivered Integrated Malaria Elimination (CIME) model induction training

|  |  | Hlegu | Kunggyangone | Taikkyi | Total |
| --- | --- | --- | --- | --- | --- |
| Nov 15 – Nov 21 | Block 1 | 4 | - | 2 | 6 |
| Nov 29 – Dec 5 | Block 2 | 1 | 2 | 2 | 5 |
| Dec 13 – Dec 19 | Block 3 | 4 | 4 | 3 | 11 |
| Dec 27 – Jan 2 | Block 4 | 2 | 1 | 2 | 5 |
| Jan 10 – Jan 16 | Block 5 | 2 | - | 6 | 8 |
| Jan 24 – Jan 30 | Block 6 | 3 | 2 | 2 | 7 |
| Feb 7 – Feb 13 | Block 7 | 6 | 2 | 1 | 9 |
| Feb 21 – Feb 27 | Block 8 | 3 | 1 | - | 4 |
| Mar 7 – Mar 13 | Block 9 | 4 | 1 | 4 | 9 |
| Mar 21 – Mar 27 | Block 10 | 2 | 4 | 2 | 8 |
|  | **Total** | 31 | 17 | 24 | 72 |

Although 75 Integrated Community Malaria Volunteers (ICMVs) were eligible and recruited over the three townships (Hlegu – 32, Kungyangon – 18, Taikkyi – 25,) at the baseline, three ICMVs dropped out from the study after attending the training.

## Supplementary Table 2: Referral, diagnosis and treatment of suspected dengue, tuberculosis (TB), diarrhoea and RDT-negative fever cases

| **Study characteristic** | **Control period** | **Intervention period** | **Total** |
| --- | --- | --- | --- |
| *Referrals (total)* | 86 | 217 | 303 |
|  |  |  |  |
| *Dengue* |  |  |  |
| Suspected | 26 | 6 | 32 |
| Referred | 4 | 1 | 5 |
|  |  |  |  |
| *TB* |  |  |  |
| Suspected | 77 | 61 | 138 |
| Referred | 41 | 38 | 79 |
| Confirmed diagnosis | 3 | 0 | 3 |
| Directly observed treatment | 2 | 0 | 2 |
|  |  |  |  |
| *Diarrhoea* |  |  |  |
| Clinically diagnosed | 8 | 104 | 112 |
| Initially treated | 0 | 57 | 57 |
| Initially treated and referred | 0 | 17 | 17 |
| Referred | 2 | 27 | 29 |
|  |  |  |  |
| *RDT-negative fever* |  |  |  |
| Suspected | 982 | 1185 | 2167 |
| Referred | 39 | 151 | 190 |

## Supplementary Table 3: The CIME intervention and malaria blood examination by RDT (n=72 villages, n=1656 weekly measurements)

|  | ***Intention-to-treat*** | | | ***As-treated*** | | |
| --- | --- | --- | --- | --- | --- | --- |
| **Factors** | ***AIRR*** | ***95%CI*** | ***p-value*** | ***AIRR*** | ***95%CI*** | ***p-value*** |
| ***Intervention*** | | | | | | |
| Control (ICMV) | ref. | - | - | ref. | - | - |
| Intervention (CIME) | 1·23 | 1·01,1·50 | 0·036 | 1·23 | 1·01,1·49 | 0·042 |
| ***Time (week)*** | 0·99 | 0·97,1·00 | 0·071 | 0·99 | 0·97,1·00 | 0·090 |
| ***Season*** | | | | | | |
| Cool | ref. | - | - | ref. | - | - |
| Hot | 1·36 | 1·15,1·60 | <0·001 | 1·35 | 1·14,1·60 | 0·001 |
| ***Variances of the random effects*** | | | | | | |
| Village |  |  | 0·30 |  |  | 0·30 |
| Week |  |  | 0·34 |  |  | 0·34 |
| the CIME intervention |  |  | 0·12 |  |  | 0·11 |
| Covariance between the random effects for village (intercept) and the CIME intervention (slope) |  |  | -0·08 |  |  | -0·07 |

*Intention-to-treat* = intervention effect estimated as per the village block randomisation sequence specified in the trial protocol.

*As-treated* = intervention effect estimated as per the actual observed village block sequencing (n=10 villages were allocated to block sequences post randomisation that differed from the protocol).

Adjusted incidence rate ratio (AIRR), 95% confidence interval (95% CI) and p-value.

Estimates from a crossed random-effect Poisson linear mixed-effects model with random effects for cross-sectional (week) and village-specific heterogeneity in RDT testing; and village-specific heterogeneity in effect of the intervention.

## Supplementary Table 4: The CIME intervention and referral for suspected dengue, TB, diarrhoea and RDT-negative fever (n=67 villages, n=1541 weekly measurements)

|  | ***Intention-to-treat*** | | | ***As-treated*** | | |
| --- | --- | --- | --- | --- | --- | --- |
| **Factors** | ***AIRR*** | ***95%CI*** | ***p-value*** | ***AIRR*** | ***95%CI*** | ***p-value*** |
| ***Intervention*** | | | | | | |
| Control (ICMV) | ref. | - | - | ref. | - | - |
| Intervention (CIME) | 3·17 | 1·23,8·18 | 0·017 | 3·30 | 1·25,8·75 | 0·016 |
| ***Time (week)*** | 0·99 | 0·92,1·06 | 0·793 | 0·99 | 0·92,1·06 | 0·716 |
| ***Season*** | | | | | | |
| Cool | ref. | - | - | ref. | - | - |
| Hot | 0·85 | 0·31,2·32 | 0·756 | 0·87 | 0·32,2·4 | 0·788 |
| ***Variances of the random effects*** | | | | | | |
| Village |  |  | 9·10 |  |  | 8·60 |
| Week |  |  | 0·16 |  |  | 0·16 |

*Intention-to-treat* = intervention effect estimated as per the village block randomisation sequence specified in the trial protocol.

*As-treated* = intervention effect estimated as per the actual observed village block sequencing.

Adjusted incidence rate ratio (AIRR), 95% confidence interval (95% CI) and p-value.

Estimates from a crossed random-effect Poisson linear mixed-effects model with random effects for cross-sectional (week) and village-specific heterogeneity in referral.

| **Village block/sequences** | **Study period (week)** | | | | | | | | | | | | | | | | | | | | | | | | | |
| --- | --- | --- | --- | --- | --- | --- | --- | --- | --- | --- | --- | --- | --- | --- | --- | --- | --- | --- | --- | --- | --- | --- | --- | --- | --- | --- |
|  | **1** | | **2** | **3** | **4** | **5** | **6** | **7** | **8** | **9** | | **10** | **11** | **12** | **13** | **14** | **15** | **16** | **17** | **18** | **19** | **20** | **21** | **22** | **23** | **24** |
| **Village 1-7** | C | | C | T | I | I | I | I | I | I | | I | I | I | I | I | I | I | I | I | I | I | I | I | I | I |
| **Village 8-14** | C | | C | C | C | T | I | I | I | I | | I | I | I | I | I | I | I | I | I | I | I | I | I | I | I |
| **Village 15-21** | C | | C | C | C | C | C | T | I | I | | I | I | I | I | I | I | I | I | I | I | I | I | I | I | I |
| **Village 22-28** | C | | C | C | C | C | C | C | C | T | | I | I | I | I | I | I | I | I | I | I | I | I | I | I | I |
| **Village 29-35** | C | | C | C | C | C | C | C | C | C | | C | T | I | I | I | I | I | I | I | I | I | I | I | I | I |
| **Village 36-42** | C | | C | C | C | C | C | C | C | C | | C | C | C | T | I | I | I | I | I | I | I | I | I | I | I |
| **Village 43-49** | C | | C | C | C | C | C | C | C | C | | C | C | C | C | C | T | I | I | I | I | I | I | I | I | I |
| **Village 50-56** | C | | C | C | C | C | C | C | C | C | | C | C | C | C | C | C | C | T | I | I | I | I | I | I | I |
| **Village 57-63** | C | | C | C | C | C | C | C | C | C | | C | C | C | C | C | C | C | C | C | T | I | I | I | I | I |
| **Village 64-72** | C | | C | C | C | C | C | C | C | C | | C | C | C | C | C | C | C | C | C | C | C | T | I | I | I |
| C | | Control | | | | | | | | |  |  |  |  |  |  |  |  |  |  |  |  |  |  |  |  |
| T | | Training – no testing data collected | | | | | | | | |  |  |  |  |  |  |  |  |  |  |  |  |  |  |  |  |
| I | | Intervention | | | | | | | | |  |  |  |  |  |  |  |  |  |  |  |  |  |  |  |  |

## Supplementary Figure 1: Stepped- wedge cluster- randomised controlled trial design.

Villages were grouped into nine blocks/sequences of seven villages and the last tenth block/sequence of nine villages, with blocks/sequences transitioned from control (Integrated Community Malaria Volunteer, ICMV) to intervention (the CIME) condition at fortnightly intervals in random order following a four-day training and transition period. This follows an initial two-week baseline period at the start of the study period where all village clusters used the ICMV model exclusively.
